# Datasets and their influence on the development of computer assisted synthesis planning tools in the pharmaceutical domain†
†Electronic supplementary information (ESI) available. See DOI: 10.1039/c9sc04944d


**DOI:** 10.1039/c9sc04944d

**Published:** 2019-11-05

**Authors:** Amol Thakkar, Thierry Kogej, Jean-Louis Reymond, Ola Engkvist, Esben Jannik Bjerrum

**Affiliations:** a Hit Discovery , Discovery Sciences, R&D , AstraZeneca , Gothenburg , Sweden . Email: esben.bjerrum@astrazeneca.com; b Department of Chemistry and Biochemistry , University of Bern , Bern , Switzerland . Email: amol.thakkar@dcb.unibe.ch

## Abstract

Computer Assisted Synthesis Planning (CASP) has gained considerable interest as of late. Herein we investigate a template-based retrosynthetic planning tool, trained on a variety of datasets consisting of up to 17.5 million reactions. We demonstrate that models trained on datasets such as internal Electronic Laboratory Notebooks (ELN), and the publicly available United States Patent Office (USPTO) extracts, are sufficient for the prediction of full synthetic routes to compounds of interest in medicinal chemistry. As such we have assessed the models on 1731 compounds from 41 virtual libraries for which experimental results were known. Furthermore, we show that accuracy is a misleading metric for assessment of the policy network, and propose that the number of successfully applied templates, in conjunction with the overall ability to generate full synthetic routes be examined instead. To this end we found that the specificity of the templates comes at the cost of generalizability, and overall model performance. This is supplemented by a comparison of the underlying datasets and their corresponding models.

## Abstract 2

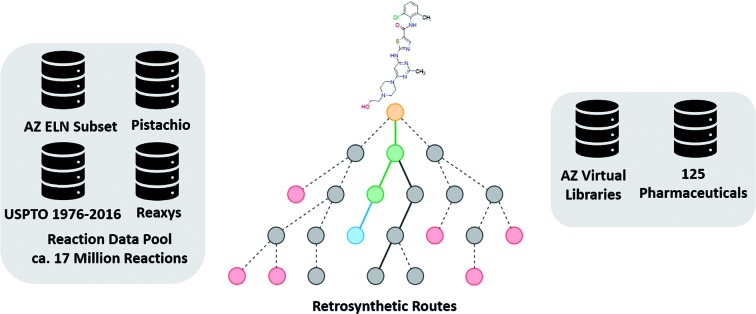
Computer Assisted Synthesis Planning (CASP), datasets and their performance.

## Introduction

Developments in computer assisted synthesis planning (CASP), specifically retrosynthetic analysis have gained considerable interest in recent years.^1^ The resurgence of artificial intelligence (AI) in computer aided drug design (CADD) has driven the shift from more traditional expert systems, built around a manually encoded set of reactions as templates,^2^ to data-driven approaches.^3^,^4^ Recent successes have been reported coupling neural networks to Monte-Carlo tree search (MCTS),^3^ and within reinforcement learning frameworks,^5^ deviating from more traditional expert systems.^2^,^6^–^12^ Their ability to rationalize a set of promising synthetic routes from reaction data, has been realized in the framework of Design, Make, Test, Analyze (DMTA) cycles, in which they have played an integral role for coupling to automation platforms.^4^ However, despite recent achievements in the field to advance predictive capability, little attention has been paid to the underlying datasets, the size of the dataset required, an assessment criteria specific to the template prioritization method and overall model performance.^13^

Retrosynthetic planning or analysis refers to the technique used by chemists to recursively deconstruct a compound into its simpler precursors, until a set of known or commercially available building blocks is reached.^14^ After an initial pattern recognition step, a chemist works in the reverse direction, using a knowledge-base of synthetic transformations (‘synthetic tool-box’) obtained through years of experience and exposure to a variety of both successful and failed chemistry,^15^,^16^ to intuitively identify and prioritize a promising set of forward transformations required to synthesize a given compound. To complement this process, computer assisted synthesis planning (CASP) tools are desired that can rapidly consider a vast body of chemical knowledge, effectively prioritize a set of reactions, and develop synthesis plans that can be tailored for the domain in which they will be applied. These have been reviewed extensively elsewhere.^1^,^9^–^12^,^17^–^21^ With the rise of automation,^4^,^22^,^23^ *de novo* design,^24^ and more extensive virtual libraries,^25^ such a tool has the added requirement that it must be able to pre-filter compounds prior to synthesis, thus reducing experimental failure and accelerating Design, Make, Test, Analyze (DMTA) cycles prevalent in molecular design.^1^,^4^,^26^,^27^

Herein, we investigate the role of the template prioritization method and the tree search algorithm derived from the work of Segler and Waller.^3^ Template prioritization is framed as a multi-class classification problem, for which we employ a neural network which outputs the probability of applying any given template, henceforth referred to as the policy network. This constitutes the machine learning (ML) part of the process, which we couple to a search strategy and decision-making process in the form of a tree search. Together these constitute an AI driven model for retrosynthetic planning. We examine this model in the context of the underlying datasets, pooling from internal AstraZeneca ELN, publicly available USPTO,^28^ proprietary Reaxys^29^ and Pistachio data.^30^ The overlap and relations between the datasets are examined. The final model's performance is tested on a set of 1731 compounds from a set of 41 virtual libraries designed at AstraZeneca between October 2017 and January 2019, in relation to policy network accuracy, percentage of routes found, and the number of compounds synthesized experimentally. Thereby, demonstrating the potential use for such tools in DMTA cycles, and how datasets with known experimental results can be used to assess model performance and improvement of CASP tools. As such, we relate our findings of model performance to the underlying datasets. Demonstrating that models built on datasets such as internal or publicly available data can predict synthetic routes in line with the literature.

## Results and discussion

### Template specification

Templates were extracted using an adaptation of Coley *et al.*'s implementation for rule extraction,^31^ which only contain the immediate neighborhood of the reaction centers, thus do not capture the extended environment required to account for leaving and protecting groups. In addition, the algorithm failed to account for reactive species, without specification of which, the reactants would not be regenerated. This has since been corrected by Coley *et al.* in RDChiral and has been extended in this study to encompass *ca.* 75 functional and protecting groups commonly used in organic synthesis.^32^ These were determined by analysis of frequently used reactions in the underlying datasets. We found that half of the top 10 templates across all datasets, and 12% of the Pistachio dataset accounted for protections and deprotections. This value is similar across all datasets examined in this study and demonstrates the utility of protecting group strategies in organic synthesis. Furthermore, we determined that these improvements translate into the model being able to account for the extended molecular environment for the groups specified. However, whilst the model can employ protections and deprotections, their use is not necessarily strategic. Further work is required to allow the model to learn their most appropriate use and incorporate them for maximal effect into synthetic route planning. The model is also limited in that in cannot learn the form of new protecting and functional groups from additional data and is restricted to those specified.

### Reaction datasets and template coverage

**Fig. 1:**
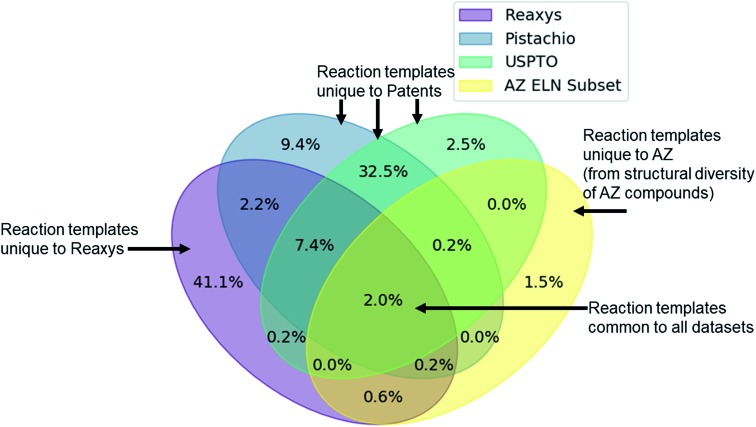
Venn diagram showing the overlap of the patent datasets (USPTO, Pistachio), Reaxys and a subset of AstraZeneca ELN data. Percentages are expressed as being part of the combined dataset. Only 2% of the extracted templates are common between all datasets, and 11.6% between Reaxys and patent data. All datasets add a unique component to the overall dataset, where the subset of AstraZeneca ELN data is the smallest contributor (4.5%) owing to the comparably lower dataset size. The two patent sets differ in content and coverage of the reaction space owing to the different time periods covered and the algorithms used for mining the data. These observations and the calculated overlap are dependent upon the template extraction strategy used, the specificity of the template (radius 1 in this case), and the subsequent procedure for the identification of duplicates/redundancies. Therefore, the percentages expressed hold true for the strategy used in this study and a template radius of 1.

Given the variety of data sources, patents (USPTO and Pistachio), literature and patents (Reaxys), and industrial data (AstraZeneca ELN), it is interesting to note that a comparable number of templates were extracted from the Reaxys and patent datasets (Table 2). However, whilst both template sets are similar in size they differ in their coverage of the reaction space as highlighted in Fig. 1. The inclusion of the Reaxys data offers a greater breadth of unique reaction templates, accounting for 41.1% of the overall combined dataset. The comparably high number of unique templates extracted from the combined patents data (32.5%), suggests that a considerable portion of patents data covered are not present in Reaxys (7.4% overlap), or that the structural components that make up the templates are unique to Reaxys. The exact differences between the patent coverage of the patent datasets (USPTO and Pistachio) and Reaxys is not clear with regards to the templates that can be obtained. Furthermore, the increased number of structural components and templates unique to the Reaxys dataset may be a residual artefact of multi-step reaction pathways. In this regard, we have filtered for all multi-step reactions, such that they have been removed from the dataset to the best of our knowledge.

The discrepancy between the two patent sets can be rationalized by the time-period over which the data was collected. The USPTO dataset accounts for reactions published up to September 2016 whereas Pistachio includes reactions until 17th Nov 2017. Further differences in the Pistachio and the public USPTO set arise from the inclusion of ChemDraw sketch data, and text-mined European patent office (EPO) patents which are included in Pistachio. The sketch data may be missing agent and condition details, as they are ‘as drawn’, and do currently not incorporate information from the accompanying text. Therefore, species that contribute a changing atom or bond may be absent and would not be incorporated in the template extraction. As this information cannot be included in the templates, the reaction is discarded, and no template is extracted.

The subset from the AstraZeneca ELN data accounts for 1.5% of unique templates. Additionally, we observe that there is a greater overlap with Reaxys than the patent data. These do not necessarily correspond to novel reactions, but rather are an artefact of the structural diversity present in the AstraZeneca collection. For instance, the synthesis of a novel lead compound could have different atomic environments around the reaction center compared to the literature or patent precedent on which it was based, thus leading to a new reaction template. Similarly, 2% of all templates are common between the datasets, thus there is a small degree of structural overlap as might be expected. These observations and the calculated overlap are dependent upon the template extraction strategy used, the specificity of the template (radius 1 in this case), and the subsequent procedure for the identification of duplicates/redundancies. Therefore, the percentages expressed hold true for the strategy used in this study and a template radius of 1. Additionally, they are an upper bound estimate for the template overlap given the template extraction strategy used in this study, and the error associated with the redundancy identification method, as not all duplicates may have been removed.

### Neural-network guided template-based retrosynthetic planning

Neural-network guided template based retrosynthetic planning methodologies were first pioneered by Segler and Waller.^3^,^33^ They trained three separate networks: an expansion policy which predicted a set of templates to be applied for a given compound, a rollout policy which predicted a stricter and more specific set of templates to be applied for a given compound, and an in-scope filter trained on positive reactions and a virtually enumerated set of negative reactions. In contrast, this study eliminates the expansion and in-scope filter policies, and focuses on a “naive” baseline retrosynthetic model using only a network inspired by that termed rollout policy by Segler and Waller.^3^

The network predicts which template to use given a compound, and a set of precursors is generated from the application of the template. This is then recursively applied to generate a retrosynthetic tree. The three primary conditions that must be fulfilled for a retrosynthetic route to be valid in this study are as follows. Firstly, there must be a template that has been extracted from the dataset which can be predicted for a given context.

Secondly, the predicted template can be successfully applied. Where successfully applied is defined as: the application of a template *in silico* that generates a set of precursors/reactants. The “success” is in reference to there being subgraph match between product and template, which enables the generation of a set of precursors, and does not reflect whether a reaction will be successful (that the reactants generated by application of the template will form the product) in the wet lab. Additionally, the set of precursors are required to be valid SMILES. It is native to the template-based approach that application of a template to the product or queried compound preserves the global structure of the compound and only alters that of the reactive site, therefore in this context it is implied that a valid SMILES also constitutes a valid set of reactants sharing the same structural features as the product. However, these are not necessarily viable precursors in the sense that they are devoid of selectivity issues and will work in the wet lab. This is a limitation we have found that is inherent to the template-based methodology and in some cases originates from the underlying dataset from which the templates were extracted, as this “error” is carried forward.

While the ultimate task is to predict synthesis that will work in the wet lab, we draw a distinction in this study by attempting to first determine what can be predicted *in silico*. To this end, we view the goal of the neural network policy as being the maximization of the number of templates that can be applied. Thereby, enumerating all possible disconnections that fall within the top 50 predicted templates for a given compound. Finally, the terminal state of a route is determined by checking if the enumerated precursors are commercially available. However, this is not to say that they are devoid of reactivity conflicts, the identification of which is left to reaction prediction models that are not implemented in this study.

### Template size and policy network accuracy

**Fig. 2:**
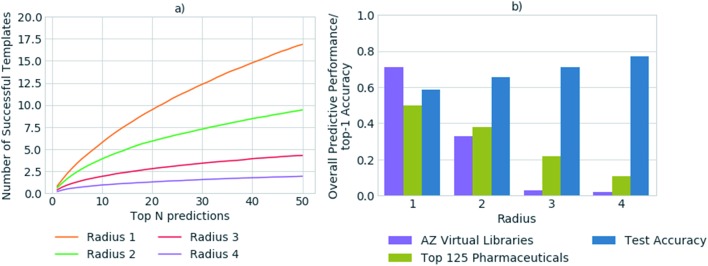
(a) The number of predicted templates that can be successfully applied to generate suitable precursors, as determined for a set of 20 000 randomly selected compounds from ChEMBL. The number of predicted templates that can be successfully applied decreases with increasing template specificity. Only *ca.* 34% of the top 50 templates are applicable on average in the best case, for the most general templates with a radius of 1. (b) Comparison of the top-1 accuracy on the test set, to overall performance with respect to the ability to generate full synthetic routes, for a set of 1731 compounds from 41 virtual libraries (AZ Virtual Libraries), and the top 125 small-molecule therapies of 2018 by sales (top 125 pharmaceuticals). The top-1 accuracy on the test set is not reflective of overall model performance and increases with template specificity. In contrast, the overall performance of the model decreases with increased template specificity as demonstrated for the virtual library and top 125 pharmaceuticals datasets.

In previous studies, accuracy has been used as a metric to gauge the network's performance for the task of retrosynthetic planning.^3^,^33^,^34^ The accuracy of the policy network reflects its ability to correctly predict a reaction template. However, for the task of retrosynthetic planning the aim is to predict several applicable templates, not just the one recorded in the dataset. Given the underlying data describes a one to one mapping of product to template and the task is to predict a one product to many templates' relationship. High accuracy values are associated with the model's ability to predict the template or reaction center from which it was originally extracted, thus overfitting the data by creating a like for like mapping to the underlying dataset. Additionally, the accuracy does not account for the applicability of the predicted template, for which we and others have found high failure rates owing to an inability to match the template substructure to the target for which it was predicted.^5^ This is illustrated in Fig. 2, whereby the increased specification of the molecular environment surrounding the reaction center (radius) leads to a higher rate of failure for its application, and translates to decreased model performance. In contrast, the test accuracy does not highlight the extent of the performance decrease, but rather increases as more of the environment surrounding the reaction center is considered, thus is misleading.

We propose that in conjunction with the accuracy, the more task- specific measure of the number of applicable templates be used for policy assessment, and a more holistic view be taken of overall model performance. In all datasets examined, on average less than 1% of all templates were applicable for any given compound. Whereby, only *ca.* 0.00035% of all templates were applicable and in the top 50 templates prioritized by the network for any given compound. Increasing template specificity further reduces the number of templates that can be applied in a given context. Therefore, to balance specificity with generalizability we propose that templates considering the reaction center and the first degree nearest neighbors be used, in conjunction with the specification of a variety of functional and protecting groups, to maintain chemical integrity.

### The effect of template library size on performance

**Fig. 3:**
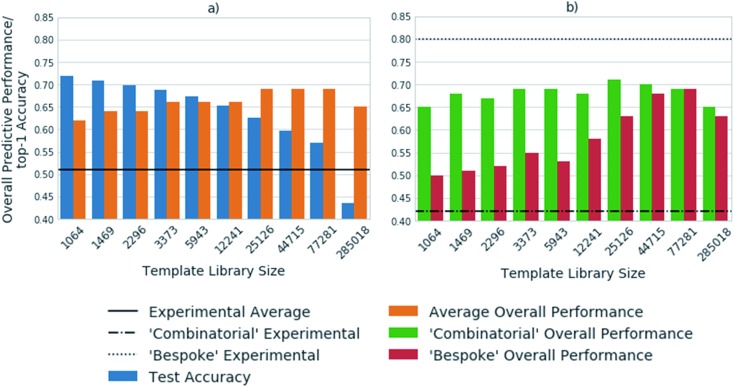
The template libraries were obtained by filtering the USPTO dataset for templates occurring a minimum of 1, 2, 3, 5, 10, 20, 35, 50, 75, and 100 times. A model was trained on each library and the results are shown for: (a) the top-1 accuracy on the test set, as compared to the overall performance. The overall performance is with respect to the ability to predict full synthetic routes to a set of 1731 compounds from 41 virtual libraries designed at AstraZeneca. The experimental average refers to the percentage of compounds synthesized out of those sent for synthesis after refinement of the virtual library. The accuracy decreases with increasing template library size, whereas the overall predictive performance increases up to a library size of the 77 281 most frequently occurring reactions. (b) The virtual library set can be further broken down into libraries designed using a ‘combinatorial’ approach, and a broader set of reactions using more ‘bespoke’ chemistry. The overall model performance increases marginally for the ‘combinatorial’ libraries with increasing template library size. Whereas, the libraries requiring more ‘bespoke’ chemistry for their synthesis benefit from the inclusion of additional reactions.

Fig. 3 shows the top-1 accuracy computed for the hold out test set for a range of library sizes using templates obtained from the USPTO dataset, as compared to the ability to predict full synthetic routes to 1731 compounds in a series of 41 virtual libraries designed at AstraZeneca. We observed that the accuracy decreases with increasing template library size, where the size of the template library reflects the top N templates in the USPTO dataset. In comparison the average predictive ability of the model increases, reflecting a more task specific measure of model performance. Where predictive ability refers to the ability of the baseline retrosynthetic model (policy network combined with tree search) to generate a retrosynthetic route. In this context the predicted route is not assessed for ‘quality’ by use of more powerful reaction prediction models,^35^ or comparison to existing literature in an automatic fashion, but rather is a reflection of whether a retrosynthetic route can be proposed *in silico* from reaction datasets.

Of note is the increasing difference between the accuracy and overall predictive performance as the library size increases. Whilst the test accuracies have been measured for a baseline template-based CASP tool, template-free models are also prone to misleading accuracy values. In both cases the task is to predict a series of viable outcomes, however the accuracy reflects the ability to predict the ‘ground truth’ from the underlying dataset, which inherently accounts for only one ‘true’ value, thus is partially known. In a similar work, Segler and Waller used the top 1, 10 and 50 accuracies to gauge the performance of their network, and showed that a model trained on 17 134 rules extracted from Reaxys, covering 52% of the dataset, was able to predict the reaction center with accuracies of 50.1%, 89.1%, and 96% respectively.^3^ In an extension of the work considering only single step reactions Baylon *et. al.* reported an accuracy of 81% on 129 rules compared to 83% on 137 rules by Segler and Waller.^33^,^34^ However, we have found that accuracy can be misleading when used for the assessment of overall model performance as shown in Fig. 2 and 3, and specifically for the assessment of whether the network is able to correctly predict applicable reaction templates for single step reactions.

The virtual library set can be further broken down into libraries designed using a ‘combinatorial’ approach, and a broader set of reactions using more ‘bespoke’ chemistry, which covers the reaction space more extensively. This enabled consideration of domain dependency with respect to template library size. We found that virtual libraries designed using a combinatorial approach benefited marginally from increasing the template library size. With the 1064 most frequently occurring templates in the USPTO dataset, routes could be found for 65% of the compounds in the virtual libraries designed using a combinatorial approach. This increased to a maximum of 72% when the 25 126 most frequently occurring templates were used. This is in line with what would be expected, as combinatorial libraries employ frequently used and robust reactions in their design.

In contrast, route predictions for libraries designed with a broader range of chemistry in mind, denoted ‘bespoke’, benefit from a larger template library size which covers the reaction space more extensively. Using the 1064 most frequently occurring templates in the USPTO dataset, the model predicted synthetic routes to 50% of the compounds in the ‘bespoke’ library, increasing by 19% to a maximal value of 69% when using 77 281 reaction templates. This alludes to the point that increasing the number of templates increases the chemical diversity of the templates, thus more synthetic routes can be found than with smaller template library sets. The increase in diversity of the templates originates from the fact that no two templates are the same, as they account for different sub-structural patterns. Increasing the template library size, also increases the probability of finding a sub-structural match to the product to which the template is applied. On the other hand, the ‘combinatorial’ libraries are less diverse, arising from the fact that a limited number of reactions were used to make them. Therefore, templates matching sub-structural patterns occurring within ‘combinatorial libraries’ are also limited. There is a balance between the number of reaction templates and the reaction space they represent, which is specific to the domain in which the tool is applied. However, increasing the number of reaction templates also introduces noise. This can be seen in Fig. 3, where the overall predictive performance falls by 4% and 6% for the ‘combinatorial’ and ‘bespoke’ libraries respectively, when increasing the template library size from 77 281 to 285 018 reaction templates. Furthermore, increasing the number of reaction templates to those that occur less frequently (less than 3 times), increases the difficulty of identifying suitable templates. The increased difficulty more than offsets the increased coverage of the reaction space (Fig. 3).

Compared to the experimental results for each virtual library, we found that the model consistently over-predicted the number of compounds that could be synthesized for the ‘combinatorial’ library. Whereas, the number of compounds that could be synthesized for the ‘bespoke’ library was consistently under-predicted. This highlights that only considering the number of compounds for which routes can be predicted does not afford enough granularity for the assessment of synthetic routes, and CASP tools. For instance, it is likely the baseline retrosynthetic model examined in this study may over predict the number of compounds that can be synthesized from the ‘combinatorial’ library, because some of the predicted steps may not translate to the wet lab. Further still, the conditions required to carry out the reaction in the forward direction are not predicted by the model, nor is there any certainty that they would yield an outcome in the wet lab if predicted. This task is left to separate models that have not been implemented in this study, that attempt to predict conditions for a queried set of substrates and a given transformation.^36^

The under-prediction of retrosynthetic routes to compounds that were experimentally obtained in the ‘bespoke’ libraries, raises questions as to the coverage of the reaction space covered by the templates, and the ability of the policy network to prioritize suitable templates. Fig. 3 examines the performance for a model trained on the USPTO dataset, thus it can be envisaged, based on Fig. 1 that inclusion of the Reaxys dataset may improve the result obtained by enabling the prediction of templates missing from the USPTO data. However, as alluded to by Fig. 3, this may increase the difficulty in identifying suitable templates, therefore improvements in the policy networks may be required for a higher number of routes to be found. The number of routes suggested by this methodology will be an upper bound estimate, which will decrease as measures are taken to increase the ‘quality’ of the suggested routes through incorporation of reaction and condition prediction models.

Furthermore, the reasons for a ‘failed’ synthesis are not always known and can be dependent on the nature of the project, the skill of the chemist, and the conditions used, to name a few factors influencing the outcome of a synthesis. These factors cannot always be quantified or considered qualitatively, thus both the predictions and ‘true’ experimental results have an associated degree of uncertainty which proves difficult to measure.

### Datasets and performance

**Fig. 4:**
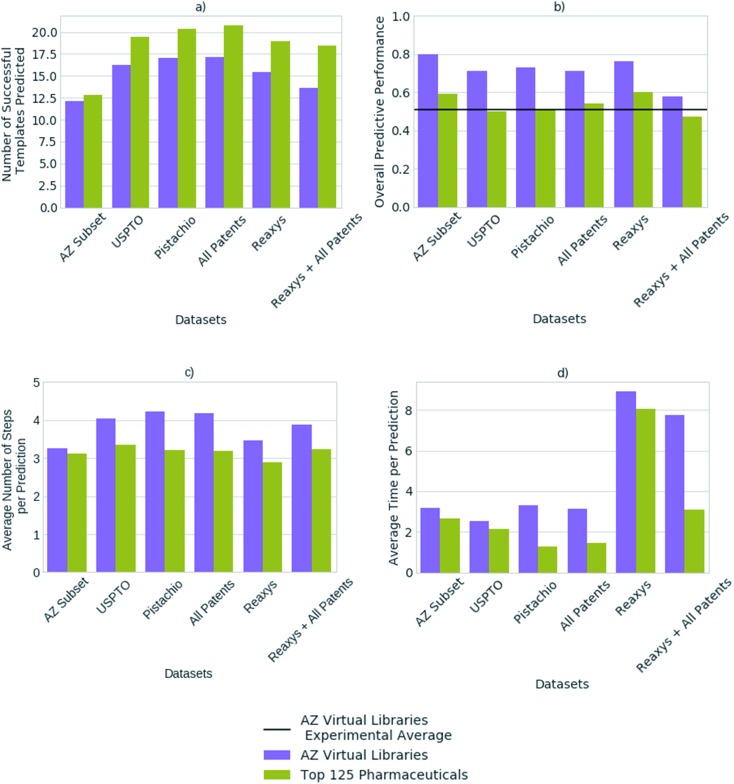
(a) The average number of successfully applied templates of the top-50 predicted templates for one-step synthesis per compound (b) the overall predictive performance with respect to the ability to generate full synthetic routes (c) the average number of steps taken per prediction per compound (d) the average time taken to predict a full synthetic route per compound, as found for each reaction dataset, for a set of 1731 compounds from 41 virtual libraries designed at AstraZeneca and the top 125 small molecule therapies by sales in 2018. The number of predicted templates that can be successfully applied for one-step synthesis does not correlate to the model's overall ability to generate full synthetic routes, when comparing between different template library sources (datasets). Whilst the model built on the subset of the AstraZeneca ELN suggests the lowest number of possible options at each step, the overall performance is comparable to, or exceeds models built on the larger reaction datasets. Thus, a model built on 4.5% of all templates considering all the datasets combined, can predict synthetic routes to compounds equally as well as the larger datasets examined.

We compared the predictive performance of models trained on each reaction dataset, and combinations thereof, on 1731 compounds from 41 virtual libraries at AstraZeneca and the top 125 small molecule therapies of 2018 (Fig. 4). The models, regardless of reaction dataset, consistently over-estimate the number of compounds that can be synthesized in the case of the virtual libraries, and under-estimate with regards to the top 125 small molecule therapies. For both cases, the average number of steps taken to synthesize a molecule is 4, however the average time taken to solve each molecule varies considerably with the dataset size (Fig. 4). The smaller datasets are faster at finding routes to a given compound (<4 seconds) owing to a smaller search space in comparison to the larger search spaces associated with the larger datasets (Pistachio and Reaxys). The simple architecture used is not able to handle the large search space and is biased towards frequently occurring reactions, which are augmented by the additional data in the larger sets. In the case of the top pharmaceutical compounds, the lower predictive performance may arise from more sophisticated ring systems, and natural product like structures upon which the final compound is based. Reactions of this nature are not prioritized by the network as they are infrequent, thus become difficult to separate from the noise. Whereas predictive performance on the virtual library dataset is higher than that for the top 125 small molecule therapies of 2018 across all datasets, as they make use of the most frequently employed reactions.

The average number of successfully applied templates of the top 50 predicted templates for one-step synthesis per compound varies considerably across the reaction datasets examined (Fig. 4). The model built on a subset of the AstraZeneca ELN appears to be worse than the models built on other reaction datasets by this measure. However, we have found that the number of options the network suggests for one-step synthesis does not impact overall model performance in this case. Thus, as Segler and Waller suggested in a previous study examining training set size,^33^ models competitive with those built on larger reaction sets can be obtained with datasets as small as an internal ELN. The subset of the AstraZeneca ELN accounts for 4.5% of the template library obtained from a combination of all datasets examined, yet is capable of providing sufficient training data to train policy networks and resulting models which are competitive with those of larger proprietary datasets. However, we expect that this is domain specific and reflects that the subset of the AstraZeneca ELN is tailored to the medicinal chemistry domain in comparison to the patent and Reaxys datasets, which are more extensive in their converage (Fig. 1). This further demonstrates that there is a balance between the type of chemistry covered by the template library set, and the size of the template library. An optimal set would be domain specific, and cover enough examples of sufficient diversity, that the output space would be managable by the policy network. In the current approach we have found that as the dataset size increases, so does the output space of the policy network (Table 2). This increases the time taken to train the network, and makes it increasingly difficult for the network to prioritize appropriate reactions as seen when increasing template library size in Fig. 3.

Previous studies have demonstrated that models built on the USPTO dataset, can predict one-step synthesis. We show that despite the seemingly lower amount of data in the USPTO dataset compared to Reaxys (Table 2). The USPTO dataset accounts for 44.8% of the template library obtained from a combination of all datasets examined, in comparison to 53.7% which comes from Reaxys. Whilst there is a 8.9% difference and the coverage of the reaction space that the templates encode varies (Fig. 1), this does not appear to be a limiting factor for route prediction in the medicinal chemistry domain. Fig. 4 shows that the model trained on Reaxys marginally outperforms that trained on the USPTO dataset, at the expense of longer prediction times. Furthermore, we show that as the size of the dataset increases to a combination of both Reaxys and the combined patents data (USPTO and Pistachio), the overall performance of the model decreases with regards to both time and number of routes identified. This may reflect the decrease in performance observed in Fig. 3b, whereby increasing the number of templates increased the difficulty for the network to prioritize suitable templates.

We noted that the fingerprint size used to encode the product had a marginal effect on the ability of the model to predict full synthetic routes for the internal virtual library dataset (ESI†). In addition, we found that increasing the size of the stock library to include the ACD catalogue, increased the ability of the model to predict full synthetic routes to compounds in the virtual library. For both the ‘Combinatorial’ and ‘Bespoke’ libraries, the model was able to reduce the average time taken to predict full synthetic routes with the ACD catalogue, as well as reduce the average number of steps by one. The reduction in the average number of steps is more pronounced for the ‘Bespoke’ libraries, whereby it is consistent over both the USPTO and Reaxys datasets. This is in comparison to the ‘Combinatorial’ libraries whereby the reduction in the number of steps is not observed for the combined Reaxys and patent data (ESI†).

### Comparison of test and reaction datasets

Fig. 4 compared the performance of models built on a range of reaction datasets with two compound sets. A set of 1731 compounds obtained from internal AstraZeneca virtual libraries, and a set of the top 125 pharmaceutical compounds by sales in 2018. The former AstraZeneca virtual libraries can be viewed as general medicinal chemistry targets, given that there is no or little overlap with the reaction datasets (Table 1), to which the algorithm is able to generalize as shown in Fig. 4. Whereas, the top 125 pharmaceuticals are well-known targets in the training domain, given the much greater overlap with the underlying datasets (Table 1).

**Table 1:** Percentage overlap of compounds in each of two compound datasets, AZ virtual libraries and top 125 pharmaceutical compounds by sales in 2018, with those reported as products in each of the reaction datasets. As expected, the top 125 pharmaceuticals have a much greater overlap with the products in each of the reaction datasets in comparison to the AZ virtual library compounds. This is because they are patented compounds with a literature precedence where both the patent and literature examples predate the most recent timepoints in the underlying dataset. Furthermore, the AZ virtual library compounds do not overlap with the literature and patent datasets and lie outside the training data

Dataset	AZ virtual libraries (%)	Top 125 pharmaceuticals (%)
USPTO 1976–2016	0	47
Pistachio Nov 2017	0	58
Combined patents	0	58
Reaxys	0	70
Reaxys + patents	0	78
AZ ELN subset	2	4

We found that the baseline retrosynthetic model examined in this study can generate retrosynthetic routes for compounds outside it's training domain. While these routes may not necessarily be feasible in the wet lab, they can be viewed as ideas upon which a trained chemist can build. Alternatively, the algorithm may help to identify building blocks and precursors to a target compound that were previously not considered. In this regard, the quality of the retrosynthetic routes generated has not been assessed and is left to manual inspection.

### Exemplary synthetic routes

**Fig. 5:**
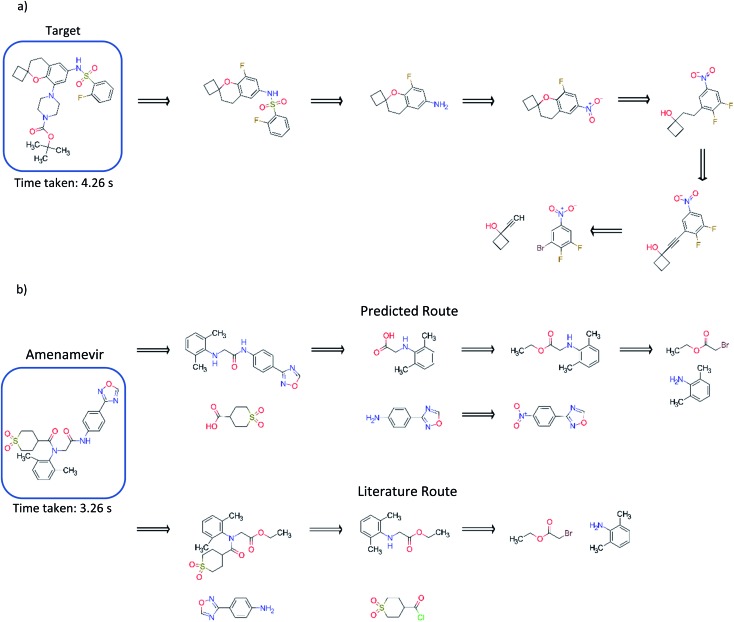
(a) Comparison to the exemplary synthesis shown by Segler and Waller.^3^ The model trained on the USPTO dataset, finds an alternative route to that in the previous study, and finds synthetic routes to the target compound in 4.26 seconds. The model can prioritize and apply ring formations as demonstrated in step 4. (b) Comparison of the route found by the model trained on the USPTO dataset with the literature route for Amenamevir.^37^ The model can suggest a route comparable to the literature, differing in the sequence of steps and using similar reactions to those in the literature. The predicted route is found in 3.26 seconds.

Comparison to existing literature in the domain showed that the model trained solely on the USPTO dataset was competitive with that reported in the literature (Fig. 5), and was able to find a route to the target compound in 4.26 seconds.^3^ This was also observed for models trained on the subset of the AZ ELN, Pistachio and Reaxys datasets. We found that the model was able to suggest an alternative route in addition to that reported, involving a ring formation (Fig. 5). Furthermore, we show that the model can predict routes to the top 125 pharmaceutical products, where the performance is dependent on the stock set of compounds. Examples of which have been given in the ESI.† The route predicted using the model trained on the USPTO dataset to Amenamevir is compared to the literature route.^37^ Both routes vary in the order of the steps they take, with the predicted route preferring a standard amide coupling over the amide Schotten–Baumann. However, the predicted route displays reactivity conflicts as deprotonation of the amine in the second step competes with the amide coupling. A further selectivity issue is present in the first disconnection step predicted for Amenamevir, as there will be competition between the nitrogen in the secondary amine and the amide. This is not the case for the literature route due to the ordering of the steps. Selectivity issues are also observed in Fig. 5a for the last retrosynthetic step (first step in the forward synthesis) where there is competition between the –OH and alkyne C–H in the aromatic nucleophilic substitution. While we know the model to be capable of using protecting groups, these are not necessarily used in a strategic way, nor is their appropriate use always identified.

## Conclusions

We have developed and implemented a baseline retrosynthetic tool with only a single neural network, to investigate the role of the ML template prioritization method in the tree search algorithm derived from the work of Segler and Waller.^3^,^33^ We have found that models trained on datasets as small as the internal ELN (4.8% of all templates) and USPTO datasets (44.8% of all templates), are sufficient for the prediction of synthetic routes to compounds found in medicinal chemistry pipelines. Furthermore, we demonstrated the potential use for such tools in compound selection and prioritization in DMTA cycles and suggest that datasets with known experimental results can be used to assess model performance.

In addition, we demonstrate that accuracy can be a misleading measure for the performance of the policy network and final tree-search model. Thus, we propose an alternative approach to assessing the ability of the policy network to identify and maximize the number of templates that can be applied, based on the number of templates that can be successfully applied in the top N predictions, for a given context. We demonstrate that the specificity and generalizability of the extracted templates must be balanced such that, the first degree nearest neighbors to the reaction center, are used in conjunction with the specification of functional and protecting groups that are common in organic chemistry.

We have found there is a dependence between the size and content of the template library used, and the domain in which it is applied. We found that syntheses of compounds originating from combinatorial libraries could be predicted using the most frequently occurring reactions. In contrast, compounds originating from libraries requiring more complex syntheses, required an expanded template set for their successful prediction. Further work is required to make use of the broad selection of reactions available to improve the variety and complexity of routes suggested. Further investigations into the template extraction process are also required to determine their descriptive limits and how this translates into route prediction.

## Methods

### Reaction datasets and template extraction

Of the datasets used, only the United States Patent Office extracts (USPTO) ranging from the years 1976 to 2016 is publicly available.^28^ This is split into granted and applied patents and is openly available for use by the community. A subset of the AstraZeneca Electronic Notebooks (ELN) were mined (May 2019) to yield the internal proprietary dataset, considering only positive reactions, classified as those with a yield greater than 1% and having a conclusion statement. The Pistachio (2017-11-17)^30^ and Reaxys^29^ datasets are commercially available, provided by NextMove software and Elsevier respectively under licensing agreements. The Reaxys dataset was filtered for multi-step reactions to yield only the intermediate single step records for which templates were extracted. Full details of the number of reactions and unique extracted templates can be found in Table 2.

**Table 2:** Datasets used in this study and their respective sizes, given as the raw dataset size without filtering

Dataset	Dataset size	Extracted and validated	Without duplicates	Templates extracted
USPTO 1976–2016^*a*^ (ref. 28)	3 748 191	3 079 351	1 201 602	302 282
Grants^*a*^	1 808 938	1 471 088	895 436	239 895
Applications^*a*^	1 939 254	1 608 263	923 765	223 871
Pistachio Nov 2017^*b*^	6 836 027	4 897 300	1 627 792	367 488
Combined patents	10 587 618	7 976 651	1 711 330	358 307
Reaxys^*b*^	6 540 786^*d*^	5 071 074	4 571 364	361 603
Reaxys + patents	17 128 404	13 047 725	6 141 875	665 288
AZ ELN subset^*b*^ ^,^^*c*^	398 779^*d*^	254 468	207 868	30 805
**All combined**	**17 523 783**	**13 302 193**	**6 342 331**	**675 530**

^*a*^Publicly available.

^*b*^Proprietary.

^*c*^Only successful reactions have been considered.

^*d*^Values reported are those after an initial internal data curation step. Dataset size: refers to the number of reactions available as reaction SMILES before curation or subsequent filtering, unless otherwise specified. Extracted and validated: refers to the number of reactions that remain after curation, automatic template extraction, and validation of the extracted template by application to the product of the reaction from which it was extracted, to determine if the corresponding reactants can be regenerated. Duplicates were identified as identical reaction SMILES considering variations in the ordering of different entities and atom-mapping. Duplicate templates were identified in the same manner. The number of products refers to products of single step reactions, where duplicates have been removed.

All reactions were atom-mapped and classified using the commercially available Filbert and HazELNut packages (v. 3.1.8) provided by NextMove software.^38^ These were subsequently processed using RDKit and RDChiral for template extraction,^32^,^39^ in conjunction with a custom reaction class developed by the authors to facilitate reaction processing. The reactions are parsed as reaction SMILES,^40^ along with the ID linking back to the data source, and classification code or textual classification obtained from the NameRxn software.^41^ The reaction SMILES are of the form:reactant_1_·reactant_2_·reactant_*n*_ > agent_1_·agent_2_·agent_*n*_ > product_1_·product_2_·product_*n*_where the reactants, agents, and products are separated by ‘>’ and the individual non-covalently bound species represented by a ‘·’ according to the Daylight SMILES specification.^42^ The definition of reactant and agent is ambiguous, as agents may participate in the reaction and contribute mass to the products. Additionally, as the templates are extracted based on atom-mapping, only the species contributing to the product or changing during the reaction were considered in the process. Thus, we have moved all agents into the reactants to give a reaction SMILES of the form:reactant_1_·reactant_2_·reactant_*n*_·agent_1_·agent_2_·agent_*n*_ ≫ product_1_·product_2_·product_*n*_

Through string manipulations, the reaction SMILES were split into their component parts on the ‘≫’ ensuring that the number of parts did not exceed three, one for each, reactants, agents, and products. Reactions leading to more than one product, incomplete reactions (*i.e.* missing reactants or products), or reactions in which the reactants and product were equivalent were removed. Equivalence was determined by converting the reactants and products to InChI and comparing.^43^ Permutations in the ordering of reactants and products were accounted for, however this was not significant in this case as we only account for reactions with one product.

Reaction templates were extracted as SMIRKS patterns using RDChiral,^32^ which we modified to consider an additional *ca.* 70 commonly occurring functional and protecting groups as determined by an analysis of the underlying datasets and extended to commonly used protecting groups in the wider literature.^44^,^45^ These are automatically identified through a substructure search of the encoded protecting groups and included in the templates alongside the reaction center and first degree nearest neighbor atoms. The reaction center is defined as atoms and bonds that change during the reaction. Owing to the number of variations sharing the same core structure for some protecting groups *i.e.* silyl ethers, esters, but varying in alkyl chain length, we have refrained from an exhaustive encoding of all possible protective groups. Rather, we have focused on those we found to be commonly occurring in the dataset and cover the main form of the protecting group, leaving the decision of the exact form to the chemist.

**Fig. 6:**
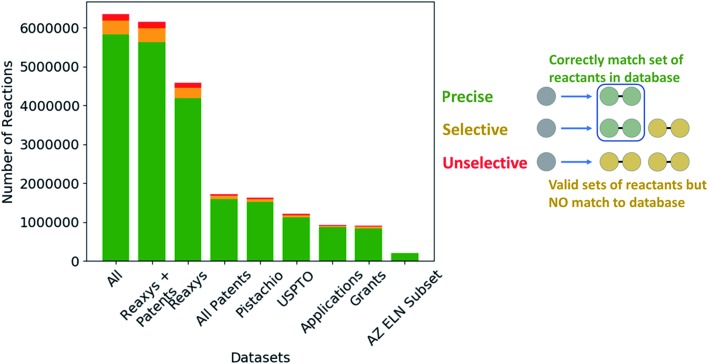
(Left) Comparison of the quality of the extracted templates across the available datasets with respect to their ability to regenerate the reactants of the reaction from which the template was extracted. (Right) Schematic of the categorization criteria used for determining the reaction templates selectivity, which we use as an initial measure of quality. The categories are defined as: (precise) the template can generate only the reactants from the reaction from which the template was extracted. (Selective) The template generates the reactants from the reaction from which the template was extracted in addition to other possible precursors that are not part of the original reaction. (Unselective) The template generates reactants that do not correspond to any of the reactants in the reaction from which the template was extracted. These may or may not be viable reactants.

The extracted templates were parsed and checked for validity in RDKit,^39^ following which the template was applied to the product of the reaction from which it was extracted to determine if an outcome could be generated. The outcomes were assessed using the definitions shown in Fig. 6, and the quality of the template extraction process quantified.

**Fig. 7:**
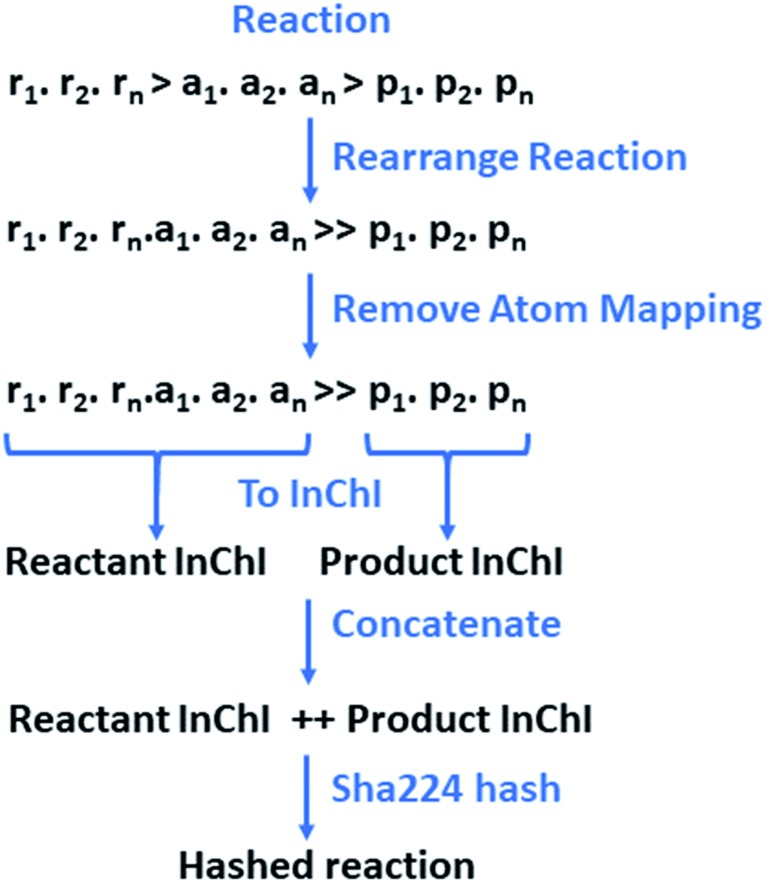
The reaction is initially rearranged to overcome the need for classification between reactants and agents, as the line is often blurred, and their definitions are often the source of debate. Atom-mapping is subsequently removed to overcome the discrepancies between toolkits, and variances in the positioning of the reactants and agents at the point of atom-mapping. The reactants and products are converted to a RDKit mol objects in without separation of the individual species. Conversion to InChI for the reactants and products respectively is carried out in RDKit.^43^,^46^ This is order invariant and overcomes the issue of having multiple SMILES representing the same molecular structure. The resulting InChIs are concatenated and hashed.

The reactions and resulting templates were hashed individually following a hashing scheme developed by the authors inspired by the reaction InChI (Fig. 7).^46^ This was also used to identify duplicate reactions and templates and can be used as an identifier for database lookups.

The datasets used in this study and their respective sizes, given as the raw dataset size without filtering are shown in Table 2. To our knowledge, the combined dataset is the largest reported to date. To enable clarity in the task specific curation process, the reduction in size through extraction and validation, followed by duplicate removal has been shown. Extraction refers to the extraction of reaction templates from the reaction SMILES,^40^ and validation refers to the application of the extracted template to the product of the reaction from which it was extracted, to determine if the corresponding reactants can be generated. Duplicates were identified as reaction SMILES consisting of identical reactants, agents, and products, using an order invariant hashing scheme accounting for variance in atom-mapping as developed by the authors. Unique reaction templates were also identified in the same manner.

The overlap of reaction templates extracted from the respective datasets was ascertained by using the in-built set methods in Python. We have observed that some of the noise associated with automatic template extraction originates from incorrect mapping, text-mining errors, and human-error from manual curation. There are several variations of these cases including, incorrect recording of functional groups, incorrect mapping of reactive components (*i.e.* substructures present in the reactive center may also be present in the solvent or reagents, for instance the incorrect mapping of an amine in both the reactant and base), accidental extension of alkyl chains, representation of catalysts and incomplete reactions, examples of which can be found in the ESI.† Whilst our approach to curation can identify such inconsistencies and disregard their associated reactions, further efforts are required to improve catalyst representation, text-mining, template SMIRKS generation and atom-mapping.

### Policy networks

Template libraries were constructed by filtering the respective dataset for templates that occurred a minimum of *N* times. In all cases duplicate reactions were removed prior to filtering. Products were represented as extended connectivity fingerprints (ECFP) with a radius of 2, using the Morgan algorithm in RDKit.^47^ Whereas, templates were represented as binarized labels in a one-*vs*-all fashion using the scikit-learn library using the ‘LabelBinarizer’.^48^ Both the input ECFP4 and output vectors were precomputed. Training, validation, and test sets were constructed as a random 90/5/5 split of the datasets, using a random state of 42, where the datasets were shuffled prior to splitting. This was conducted using the scikit-learn library.^48^

**Fig. 8:**
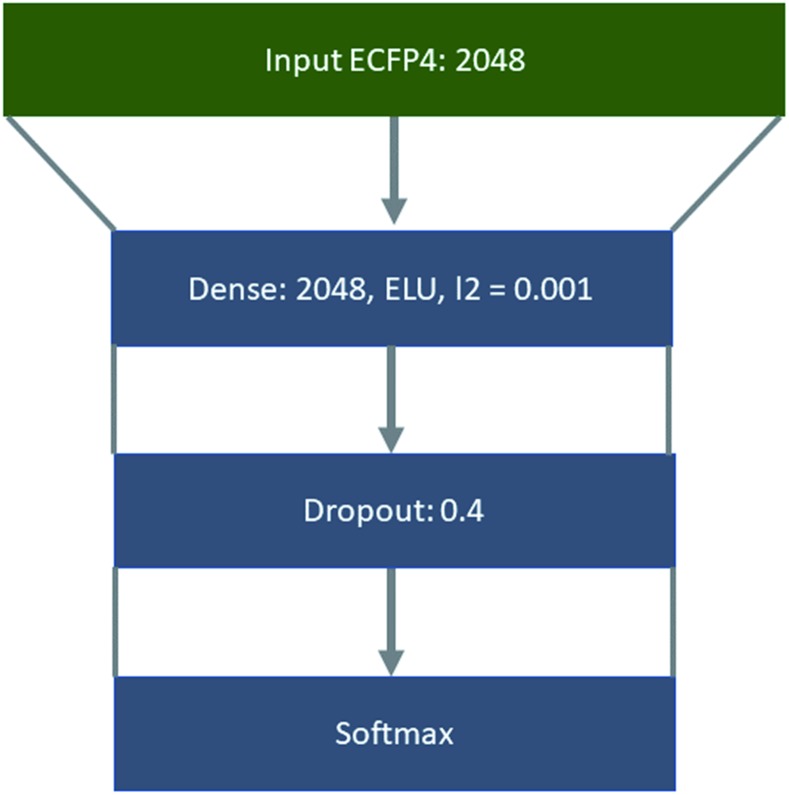
Architecture used to train the ‘rollout’ policy taking molecules represented as ECFP4 as input, through a fully connected layer of 512 nodes, ELU as the activation function, and L2 regularization set at 0.001. Followed by a dropout of 0.04 and softmax output layer.

The policy networks framed as supervised multiclass classification problems were trained using Keras^49^ with Tensorflow^50^ as the backend, the Adam optimizer with an initial learning rate of 0.001,^51^ and categorical cross entropy as the loss function (Fig. 8). The learning rate was decayed on plateau by a factor of 0.5, where the plateau was considered as no improvement of the validation loss after 5 epochs. The top 1, 5, 10, and 50 accuracies were monitored throughout the training process, and the loss on the validation set was used with early stopping (patience 10) to determine the number of epochs for which the model was trained.

### Assessing the number of successfully applied templates of the top *N* predictions

A random subset of 200 and 20 000 compounds from ChEMBL (v. 24.1)^52^ were used to assess the baseline number of applicable templates and the applicability of the top *N* templates respectively, unless otherwise stated. Salts were removed from the ChEMBL dataset using RDKit.^39^ Random subsets were drawn from the resulting dataset using a random state of 1.

The model to be assessed was loaded into Keras and the compounds to be queried converted into ECFP4 fingerprints prior to passing to the model for prediction. The top *N* predictions sorted in order of decreasing probability were used for each compound. The templates were applied to the compound in turn using RDChiral to determine if an outcome was generated. Templates leading to an outcome were classed as successful.

### Tree search with 1N-MCTS

The tree search was implemented as a simplification of the algorithm described by Segler *et al.*^3^ The MCTS algorithm was simplified with regards to the policy network. The same network was used for both the expansion and the roll-out. The prior probabilities were not used by default during the selection of leaf nodes for expansion, but the *Q* value was initialized at 0.5 and *N* at 1, as expansion counts as a first visit.

### Algorithm

The search tree is built up from nodes that contain states with current molecules of the route. The root node contains one molecule, which is the target molecule of the algorithm. Other nodes can contain states with one or more molecules. Each node is bound to others in a directed way as parent-child nodes, with actions as edges. The action is the retrosynthetic reaction performed on one of the molecules of the parent state, to yield the molecules of the child node state. The search algorithm starts with the expansion of the root node (see below).

### Selection of leaf node

In each iteration the search tree is traversed using the upper confidence bound (UCB) scores of the nodes (eqn (1)).^53^ Starting from the root node, the UCB scores of the children are calculated.1
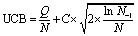
Here *Q* is the current sum of previous rewards. *N* is the number of times the child state has been visited, *N*_–1_ is the number of times the parent state has been visited. *C* is a tunable parameter balancing exploitation and exploration which was set to 1.4 by default. If the selected child is already expanded (*i.e.* has child nodes), the UCB scores of these are then calculated and the next child selected in an iterative way until an unexpanded leaf node is selected. Actions are stored at the parent level, and the child nodes are first instantiated as node objects by applying the associated action when visited (see below).

### Expansion of node

Expansion is performed by employing the expansion policy neural network for each of the molecules present in the state of the selected node. The top scored reaction templates are filtered to retain the top 50 or until a cumulative policy network score of 0.995 is reached. The possible actions (molecule + reaction) for all molecules are stored at the parent level, and vectors of associated *Q* and *N* values initialized (0.5 and 1 respectively).

The action with the highest UCB score is selected for the roll-out. In case of multiple actions sharing the largest score, random selection is performed. The child state is instantiated and added to the search tree by employing the associated reaction template to the molecule specified in the action using RDKit.^39^ In case the reaction did not give any output, the action *Q* is given a value of –10^6^, effectively preventing reselection. If no actions are available, the state is marked terminal and the state evaluated with the reward function (see below).

### Roll out

No in-scope policy was employed after the expansion phase. The roll out policy was identical to the expansion policy and thus allowed for reuse of the previous roll-outs during tree building and searching. Expansion of new child nodes during roll out is similar to the above, except the selection is done by random among the available actions. After each roll-out step the state was evaluated and the roll out stopped if either the state was solved (all compounds found in stock) or the maximum tree depth reached, or no valid actions are available.

### Reward calculation and back propagation

The reward function for the final state is then calculated (eqn (2)) and the score back propagated through the tree, updating the *Q* and *N* values of all parent states between the final state and the root state (target compound).2
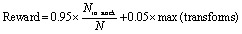

*N* is the total number of compounds in the state, *N*_in_stock_ is the number of compounds that are in stock. Transforms is the number of transforms each compound has undergone with respect to the root compound.

### Iteration and stop of search

Selection of the next leaf node to expand is then instantiated from the root node, until the maximum number of iterations or the time limit has been reached. If early stopping is wanted, the algorithm can stop if any state contains a solved state with all compounds in stock.

### Implementation

The algorithm was implemented in an object-oriented architecture, with a range of global objects for handling the search tree, the stock, the neural network predictions, settings of parameters and a logging object. The global objects were implemented using a Borg pattern that ensures singleton status and easy access though re-instantiation anywhere in the code. NetworkX was used to keep track of the parent-child relations during building of the search tree.^54^ The stock object keeps the stock as a set of InChIKeys for fast, hashed tests if compounds are contained in the stock. InChIKeys were calculated through the RDKit api for the INCHI software.^43^ Nodes and states are regular python classes, that can have several different object instances. The state object contain information about the current molecules in that state as well as the number of conversions each molecule has undergone from the root states compound. Nodes contain vectors of possible actions and child *Q* and *N* values as well as methods expansion, traversing the tree and node expansion.

### Stocks

A subset of the AstraZeneca internal catalogue and enamine building block sets were used as the stock set of compounds in all calculations unless specified. InChIKeys were computed for all compounds and duplicates removed. The subset of the AZ internal catalogue was obtained from a database dump of available compounds (January 2019) and contains 60 530 compounds. The enamine building blocks list was provided by enamine, January 2019, and consists of 162 194 compounds after preprocessing and filtering. The ACD catalogue was additionally used to provide a more extensive set of stock compounds.^55^ The compounds which had a CHIME defined where an InChIKey could be generated was extracted from ACD giving a final stock set of nearly 12.5 million compounds.

### Template library size and performance

To study the effect of library size on model performance, a filtering criterion of templates occurring a minimum of 1, 2, 3, 5, 10, 20, 35, 50, 75, and 100 times was applied to generate the appropriately sized libraries, and a policy network trained on each set.

1731 compounds spanning 41 virtual libraries designed at AstraZeneca between October 2017 and January 2019, and the top 125 small molecule therapies by sales in 2018 were used to test the algorithm.^56^ The virtual library set can be further broken down into libraries designed using a ‘combinatorial’ approach, and a broader set of reactions using more ‘bespoke’ chemistry. Knowledge of the number of compounds sent for synthesis and the number of compounds successfully synthesized was contained within the dataset. The aim was to couple the policy network to the tree search to determine for how many of the compounds a synthetic route could be predicted, and whether it was reflective of experimental results.

### Datasets and performance

Each dataset was filtered for templates occurring a minimum of three times, and a policy network trained on each set. The policy network was assessed for the number of successfully applied templates of the top *N* predictions, where *N* was 50. Subsequently the policy network was coupled to the tree search to form the overall model, which was assessed using the virtual library dataset and the top 125 small molecule therapies by sales in 2018.^56^

## Availability of data and materials

AstraZenca, Pistachio and Reaxys datasets were used with permissions. Filbert, NameRxn and HazelNut were used for atom-mapping and classification under license from NextMove software. The implementations source code will be made available at https://github.com/reymond-group/CASP-and-dataset-performance.

## Funding

Amol Thakkar is supported financially by the European Union's Horizon 2020 research and innovation program under the Marie Skłodowska-Curie grant agreement no. 676434, “Big Data in Chemistry” (“BIGCHEM,” http://bigchem.eu).

## Author contributions

Amol Thakkar and Esben Jannik Bjerrum designed and conducted the research. Thierry Kogej, Jean-Louis Reymond, and Ola Engkvist contributed ideas and provided scientific advice. Esben Jannik Bjerrum, Ola Engkvist and Jean-Louis Reymond supervised the project.

## Conflicts of interest

The authors declare that they have no competing interests.

## Supplementary Material

Supplementary information

Supplementary information
