# Supplementary material for: Datasets and their influence on the development of computer assisted synthesis planning tools in the pharmaceutical domain
Source: Chem Sci. 2019 Nov 5;11(1):154–68. doi: 10.1039/c9sc04944d (PMC7012039; doi:10.1039/c9sc04944d)

Electronic Supplementary Material (ESI) for Chemical Science.
This journal is © The Royal Society of Chemistry 2019

Supplementary Information

Datasets and their influence on the development of computer assisted synthesis planning tools in the pharmaceutical domain

Amol Thakkar,[1 2 *] Thierry Kogej,[1] Jean-Louis Reymond,[2] Ola Engkvist,[1] and Esben Jannik Bjerrum[1 *]

[1] Hit Discovery, Discovery Sciences, R&D, AstraZeneca, Gothenburg, Sweden.

[2] Department of Chemistry and Biochemistry, University of Bern, Bern, Switzerland.

*Corresponding authors: amol.thakkar@dcb.unibe.ch, esben.bjerrum@astrazeneca.com

Supporting Information 1 – Data Inconsistencies

Data inconsistencies in the USPTO dataset which highlight a wider problem with reaction data. These have been filtered out in our approach, however this is not exhaustive.

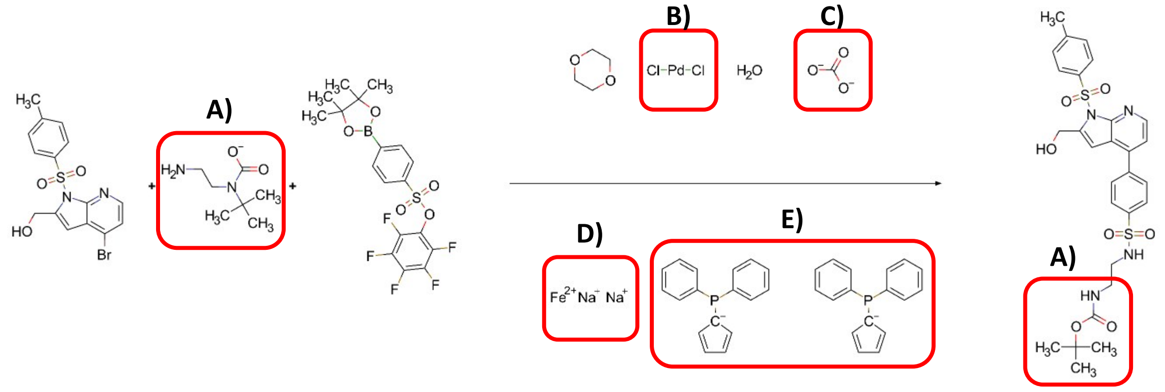

Source: US20080146606A1

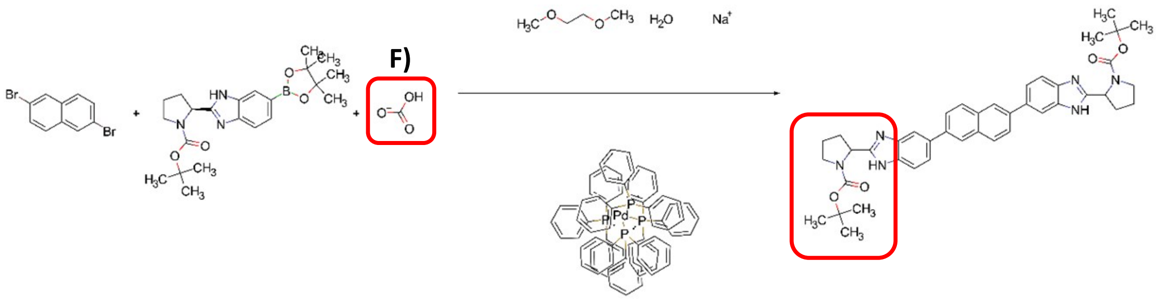

Source: US20130317213A1 [0753]

A) Incorrect recording of the Boc protecting group, frequently used in organic synthesis. In addition, the charge is not balanced in the reactants.

B) Palladium dichloride is often used to form the active catalyst in situ, however it is not clear which ligands are to be associated with the metal. A chemist can infer the active species; however, the computer must be informed which species are grouped together. This is possible using ChemAxon extended SMILES (CXSMILES), which contain information regarding the grouping of constituent parts in the reaction. For our task these do not corresponding to the changing molecular environment during the transformation, therefore are not included in the templates. As such, for the task of retrosynthesis, catalyst representation can be ignored, however is a key factor in reaction and condition prediction, so cannot be overlooked with respect to the wider field.

C) Unidentified salt lacking annotations for its utility in the shown reaction

D) Iron salt without corresponding ligands. Can be used to form a catalyst in situ or may come from a pre-formed/commercially available catalyst. It is not clear what role the species plays from a computational perspective.

E) Phosphorus based ligands which do not contribute to the changing atoms and bonds in the reaction. It is not clear to which metal the ligands bind nor their role owing to missing annotations.

F) Incorrect atom mapping arising from unbalanced reaction stoichiometry. Atoms in a species sharing substructure with the reactive species can have mislabeled atoms, thus the algorithmic extraction produces an incorrect template.

Supporting Information 2

Top 10 templates across all datasets – csv file attached to publication

Supporting Information 3

.txt file containing SMARTS patterns of the ca. 70 functional/protecting groups used

Supporting Information 4 – Example accuracy and loss curves

Accuracy and Loss curves for the model trained on the USPTO dataset, filtering for templates that occurred a minimum of three times.

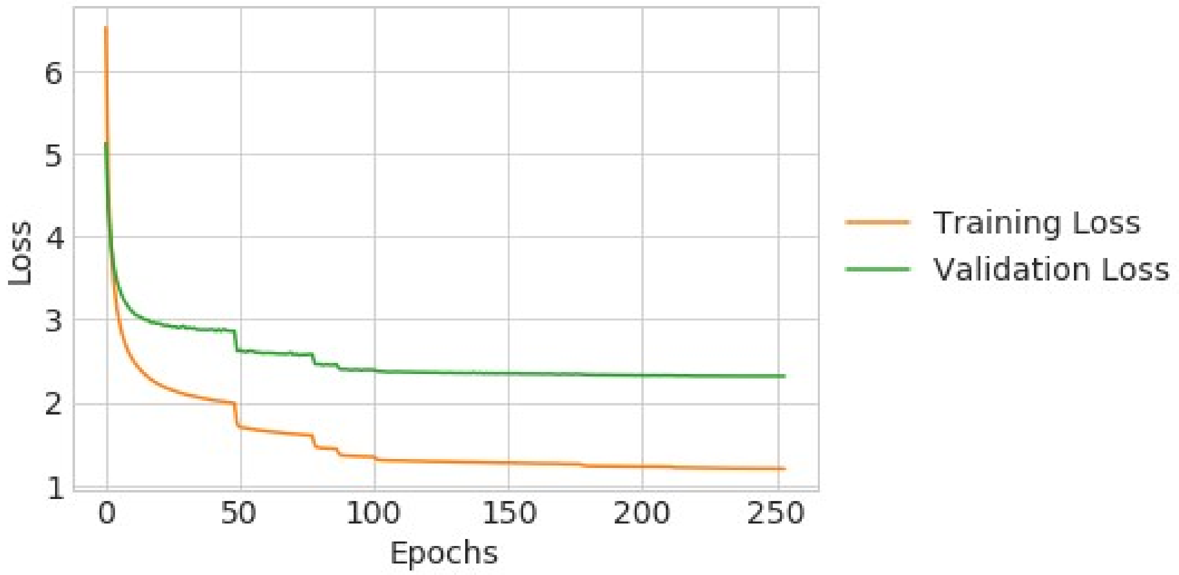

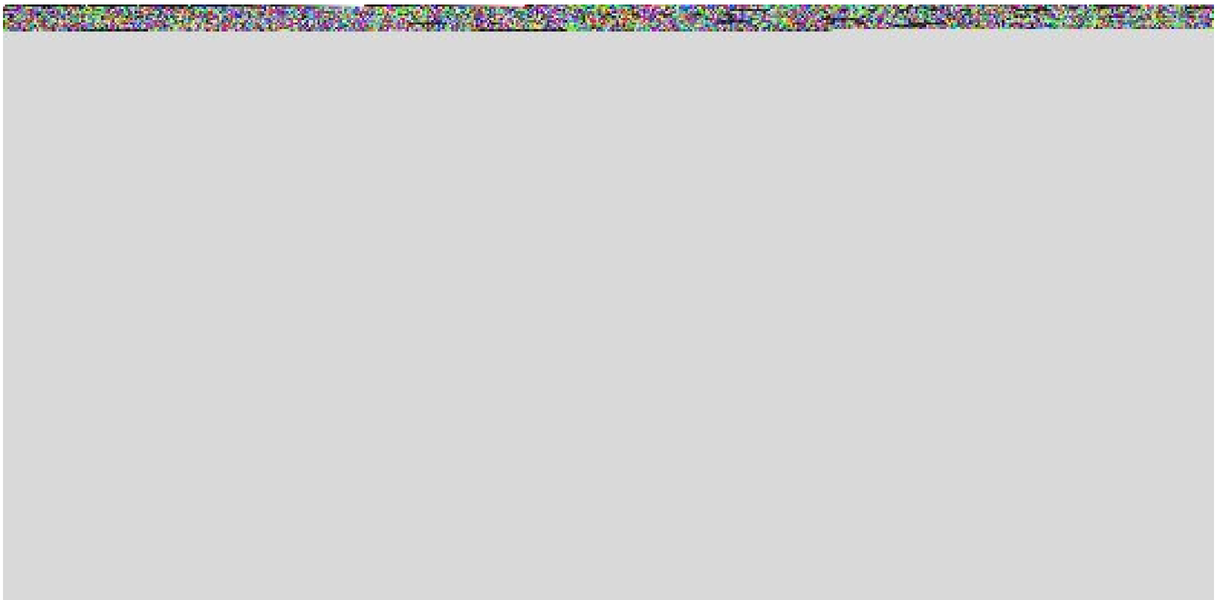

Supporting Information 5 – Input ECFP4 fingerprint size and performance

Comparison of the accuracy of models trained on different fingerprint sizes for the USPTO dataset to the iTrax virtual library dataset.

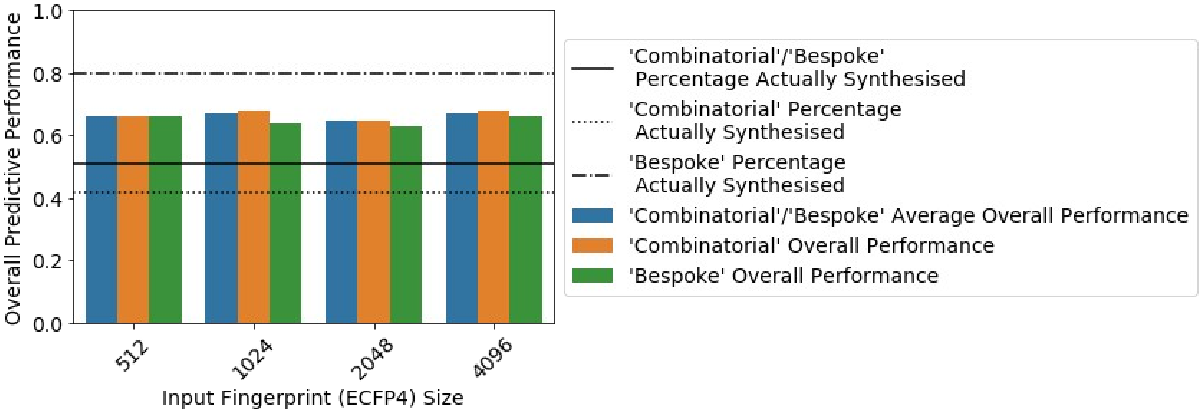

Supporting Information 6 – Performance and stock set of compounds

The performance of the model increases regardless of the dataset used when a larger stock set of compounds is used an end point. Of note is the time taken to find full synthetic routes to the target compounds, where a larger stock set performs better.

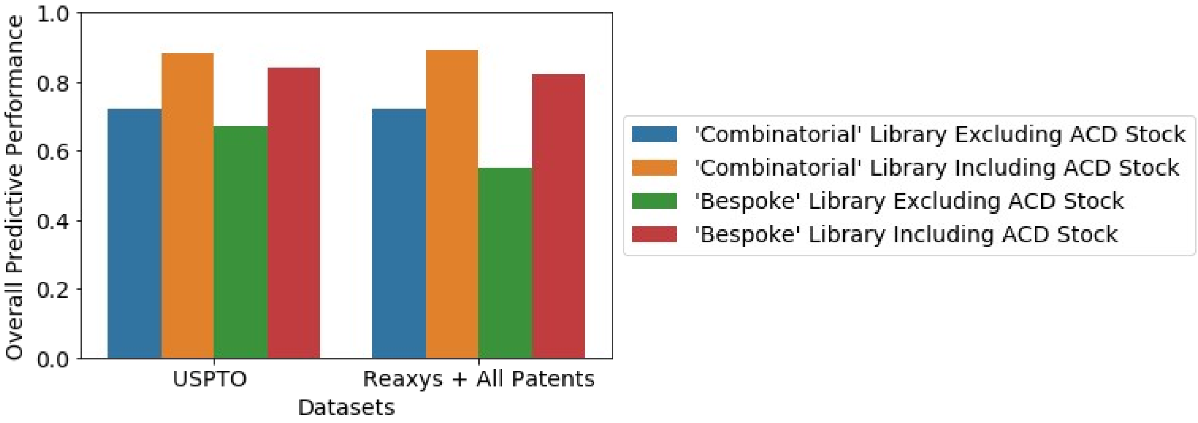

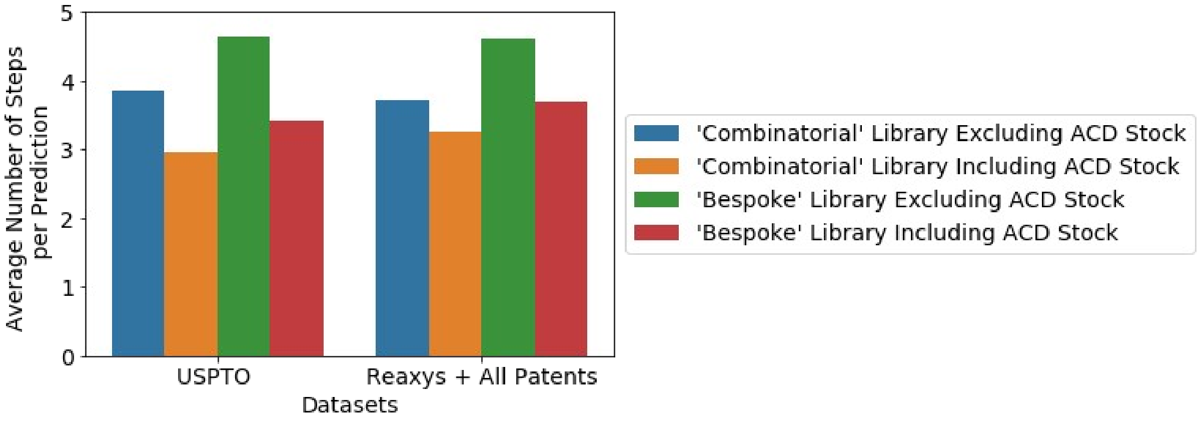

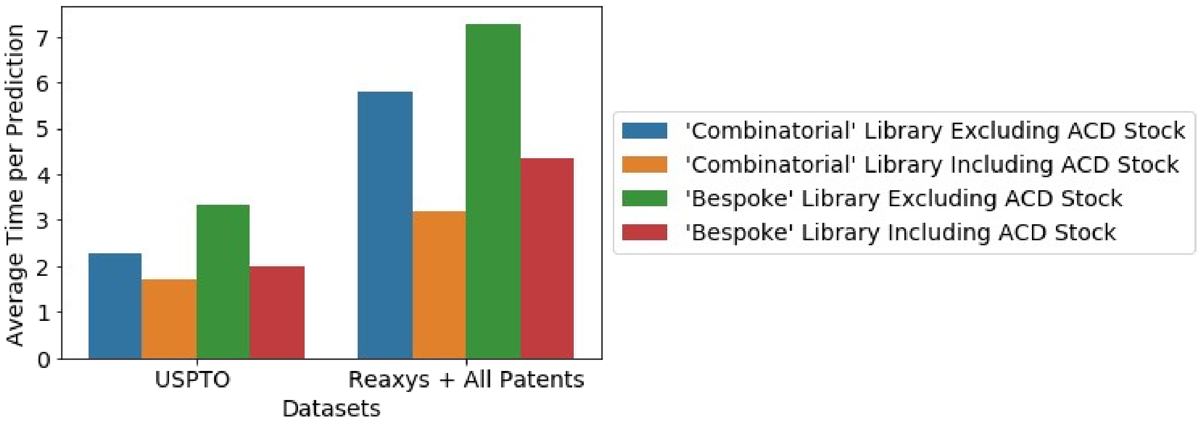

Supporting Information 7 – Exemplary Synthetic Routes

The following synthetic routes were found by the model trained on the USPTO dataset filtering for templates that occurred a minimum of three times. The AstraZeneca internal stock and Enamine building blocks were used as the stock set of compounds. The syntheses shown are for compounds in the top 125 small molecule therapies of 2018, where 47 % (59 of 125) compounds were present in the USPTO dataset. While, in some cases these have been solved faster than those that are not present, it is evident that there are selectivity conflicts and synthetic strategies employed by the algorithm that a trained chemist would avoid. These have not been filtered and the raw output of the top ranked route is given to illustrate the drawbacks of such a naïve route search yet exemplify that a naïve algorithm may in some cases still propose plausible routes.

Compound: Abiraterone Acetate

Time to solved: 0.24 seconds

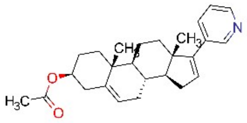

0.2407808303833008

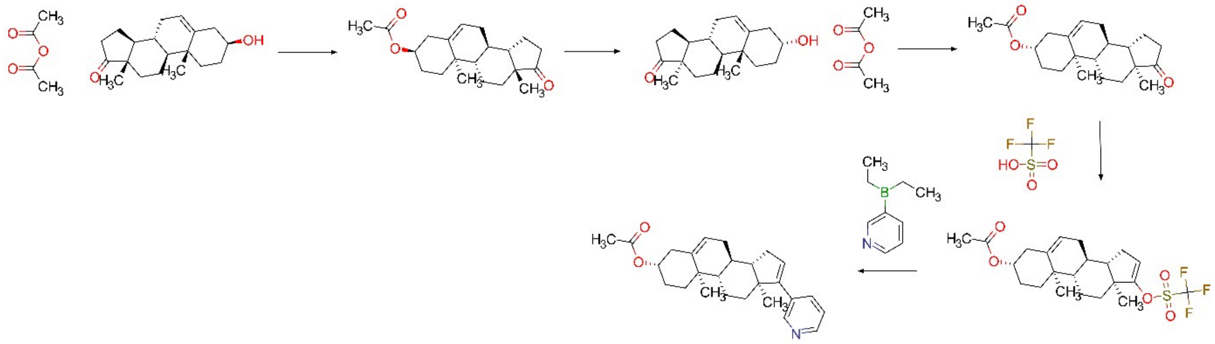

Compound: Cinacalcet

Time to solved: 0.12 seconds

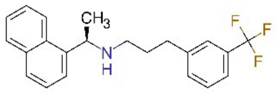

0.10924839973449707

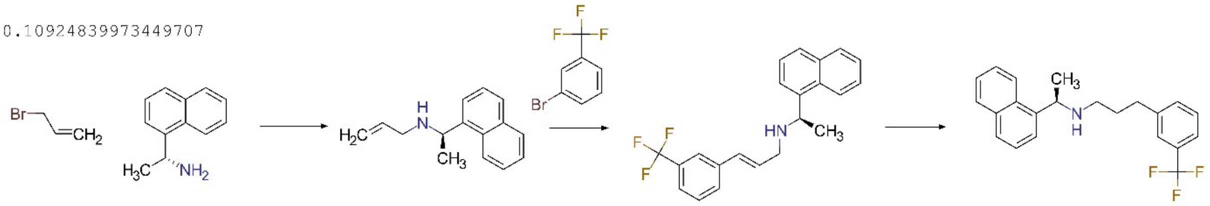

Compound: Clopidogrel

Time to solved: 7.59 seconds

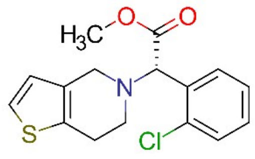

7.594855070114137

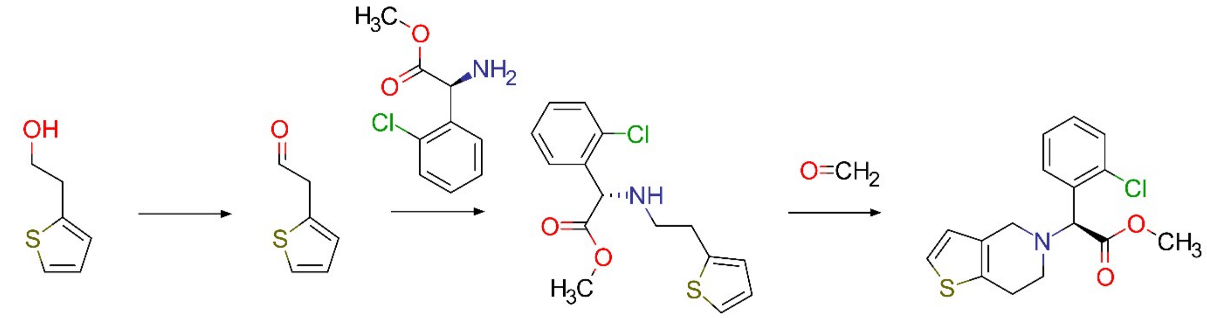

Compound: Cobicistat

Time to solved: 0.58 seconds

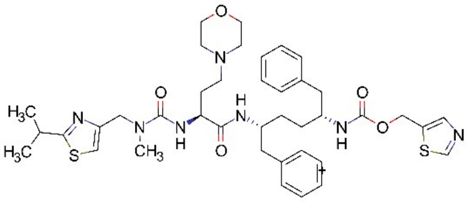

0.5831310749053955

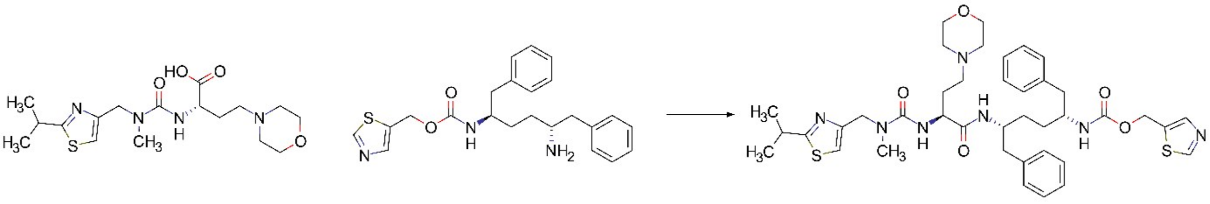

Compound: Dabigatran

Time to solved: 0.07 seconds

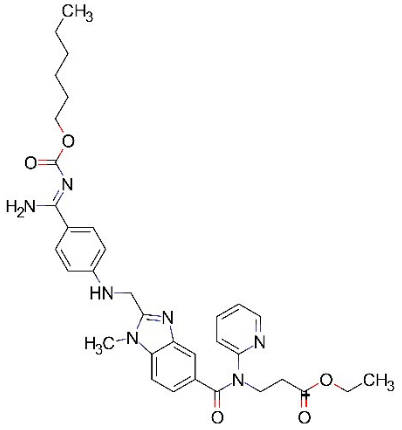

0.07490253448486328

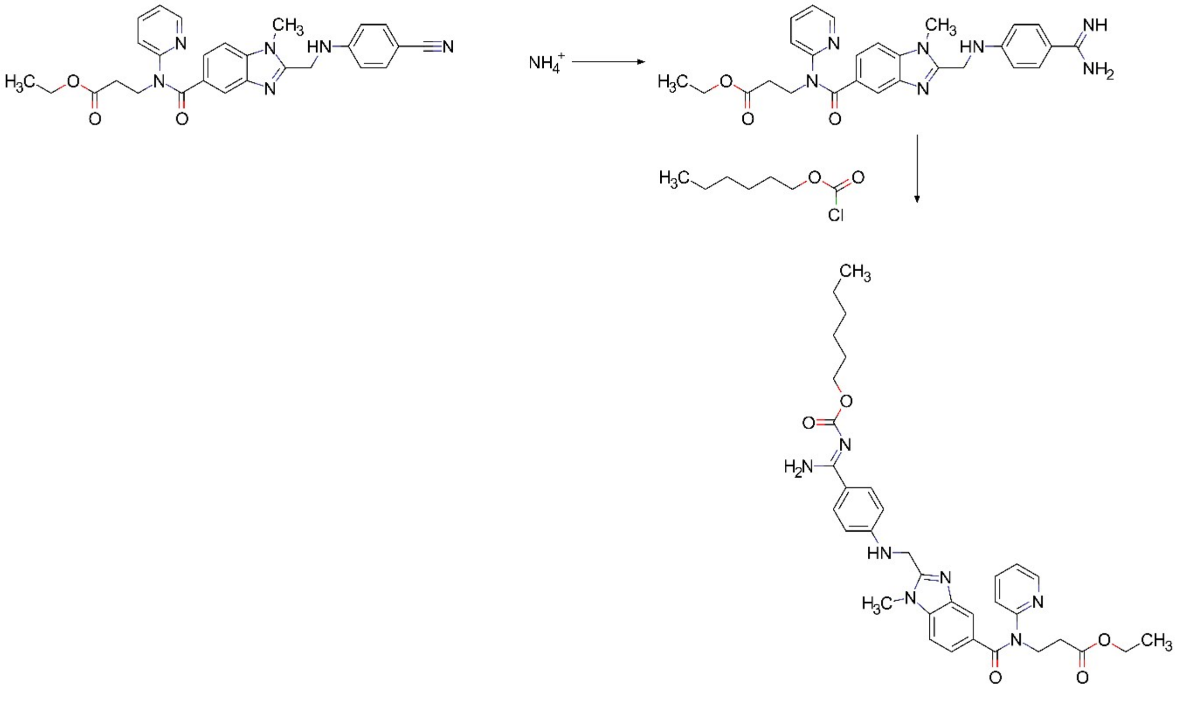

Compound: Dimethyl Fumarate

Time to solved: 1.25 seconds

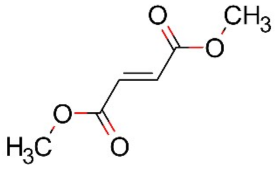

1.2493224143981934

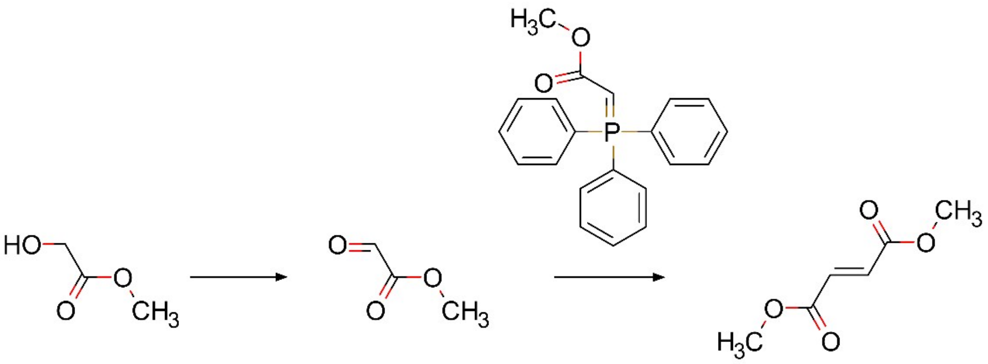

Compound: Elvitegravir

Time to solved: 13.64 seconds

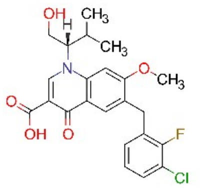

13.63359785079956

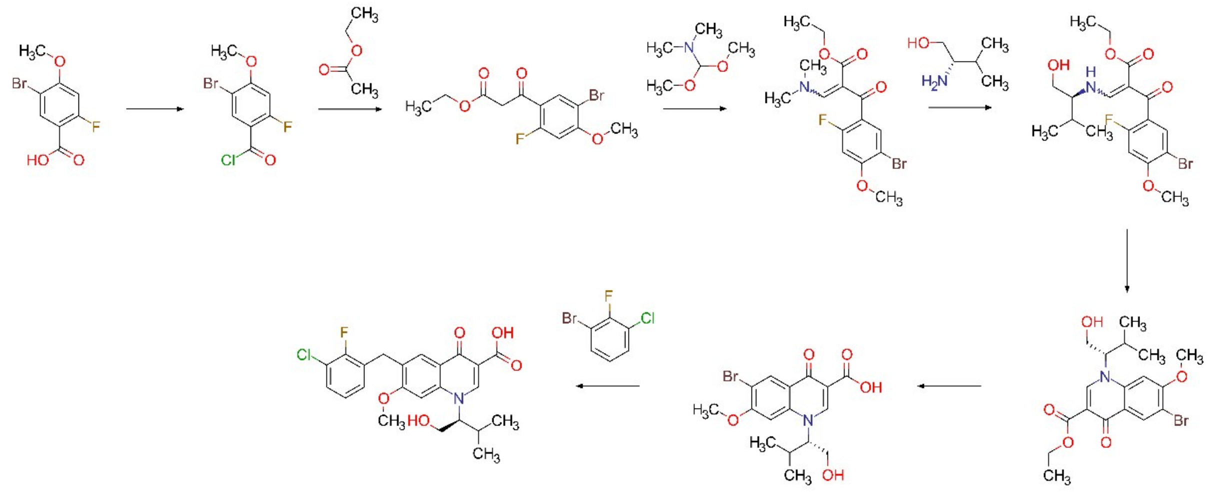

Compound: Fingolimod

Time to solved: 0.49 seconds

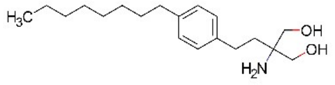

0.4869771003723145

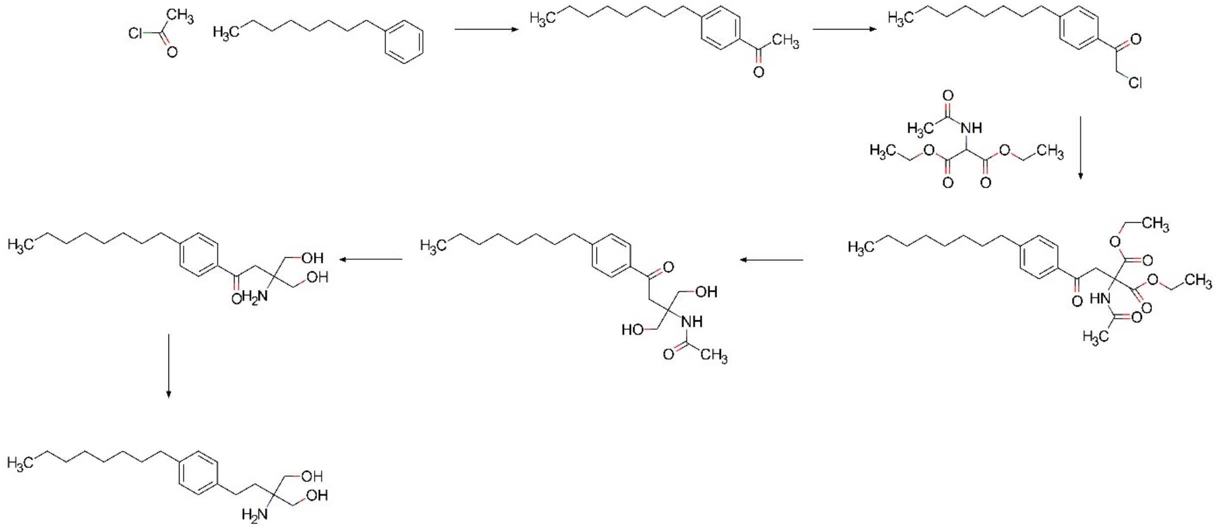

Compound: Imatinib

Time to solved: 0.21 seconds

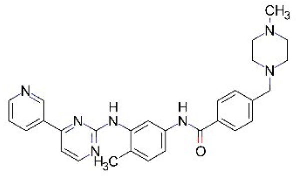

0.21118963821411133

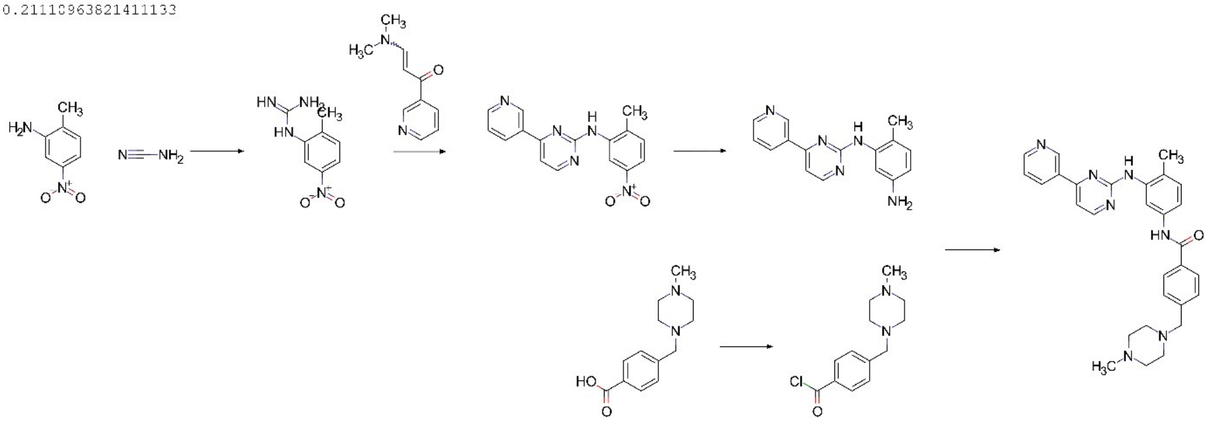

Compound: Linagliptin

Time to solved: 0.19 seconds

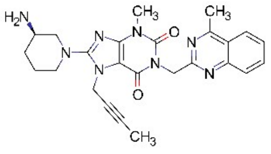

0.19151520729906494

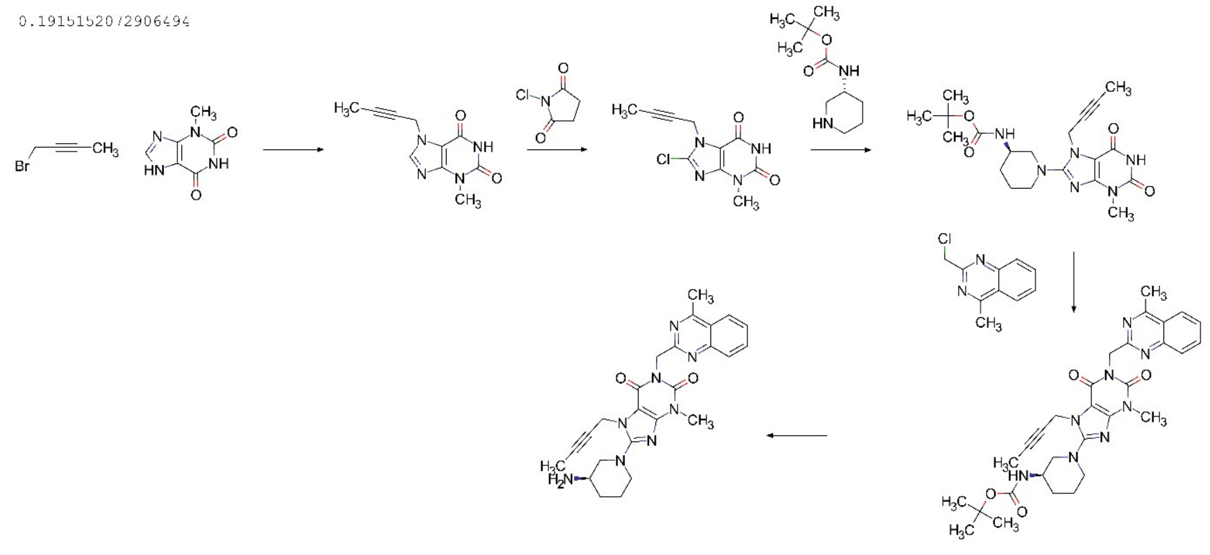

Compound: Osimertinib

Time to solved: 0.11 seconds

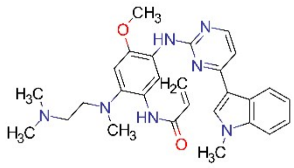

0.11084532737731934

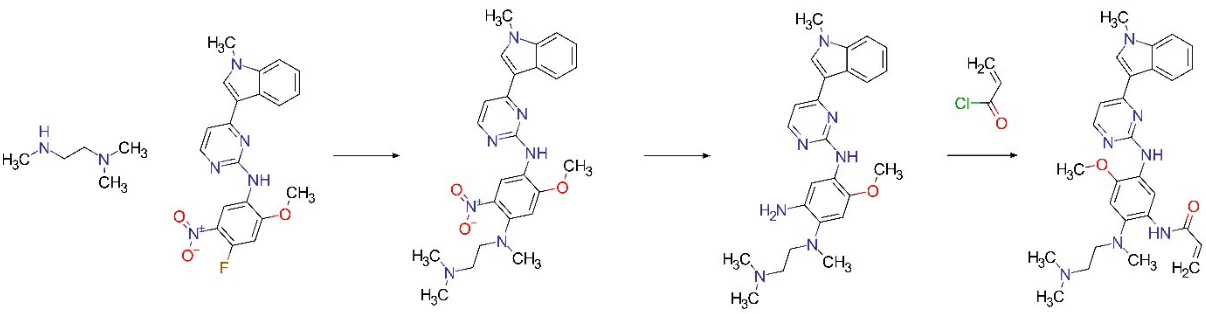

Compound: Rilpivirine

Time to solved: 0.11 seconds

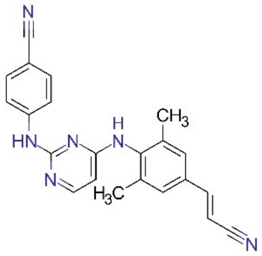

0.11122989654541016

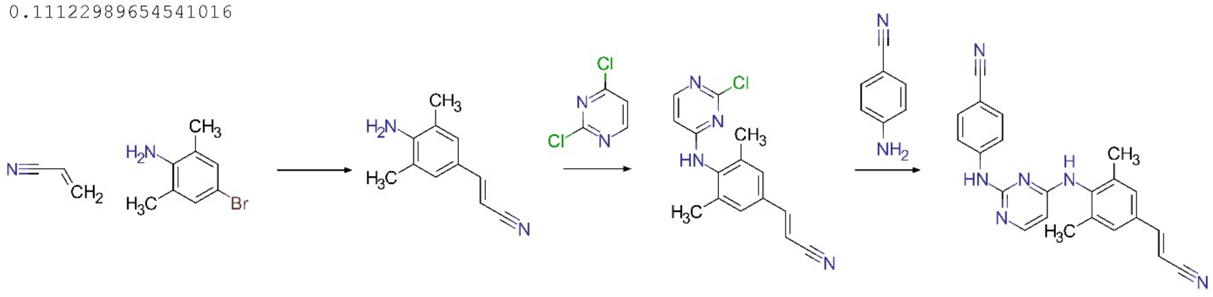

Compound: Rivaroxaban

Time to solved: 0.21 seconds

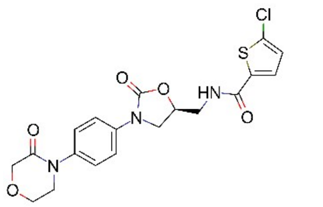

0.20984411239624026

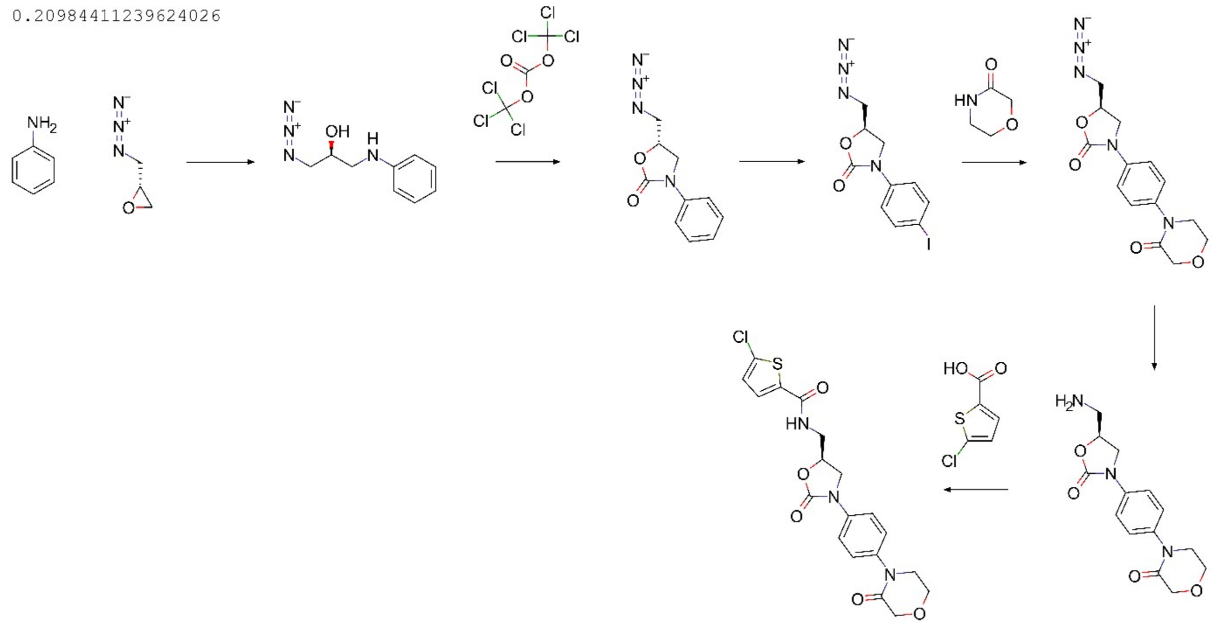

Compound: Salmeterol

Time to solved: 3.38 seconds

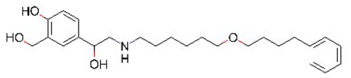

3.3805201053619385

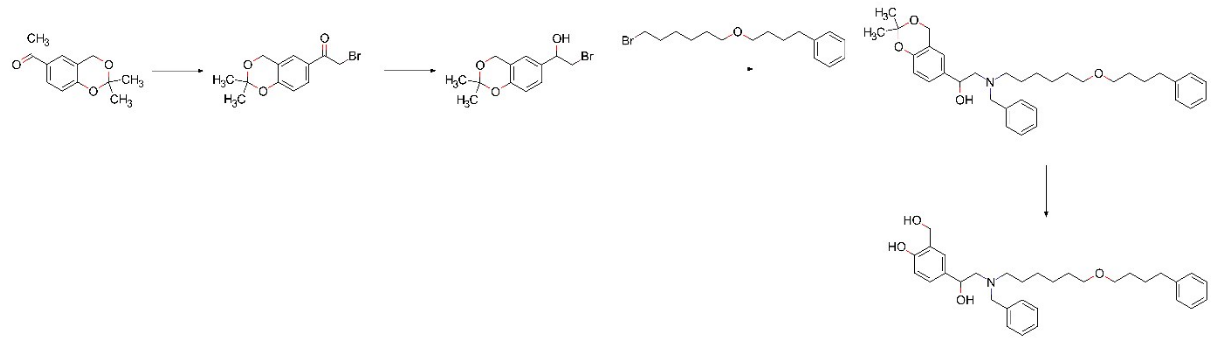

Compound: Apixaban

Unsolved: 27.10 seconds

Reason: Precursor not in stock

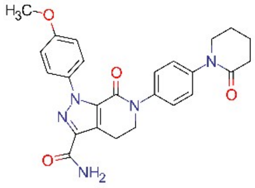

27.09695267677307

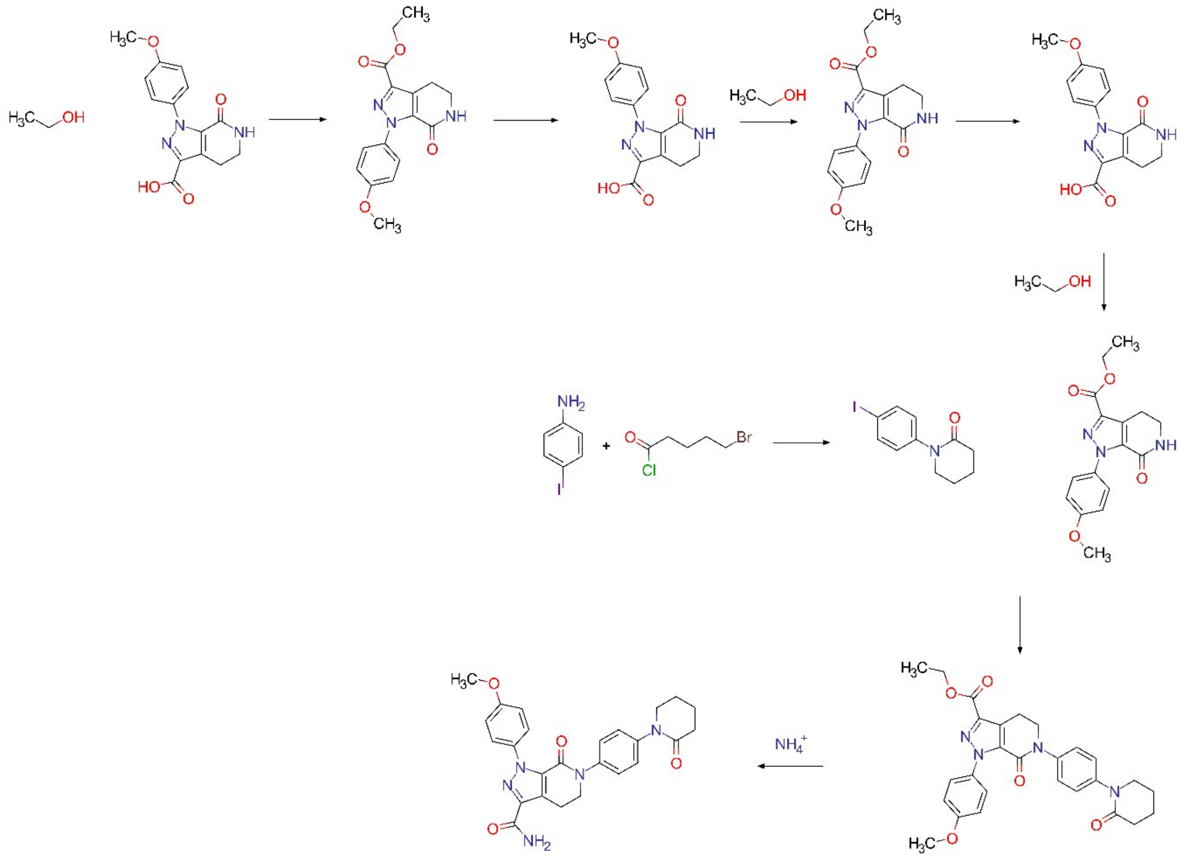

Compound: Ibrutinib

Unsolved: 37.59 seconds

Reason: Precursor not in stock

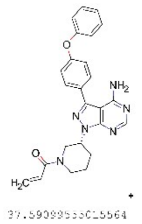

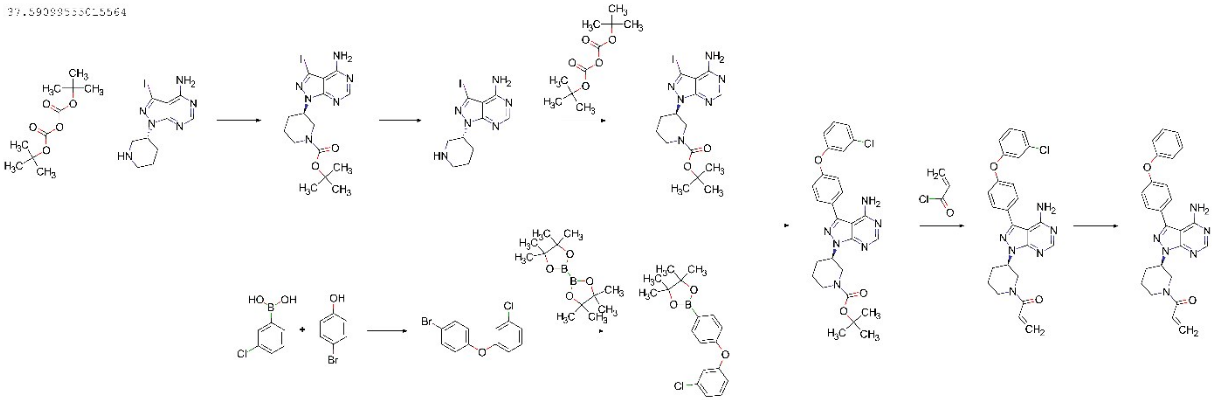

Compound: Palbociclib

Unsolved: 27.53 seconds

Reason: Precursor not in stock

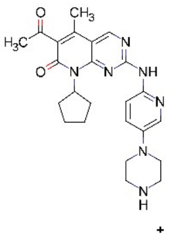

27.53420639038086

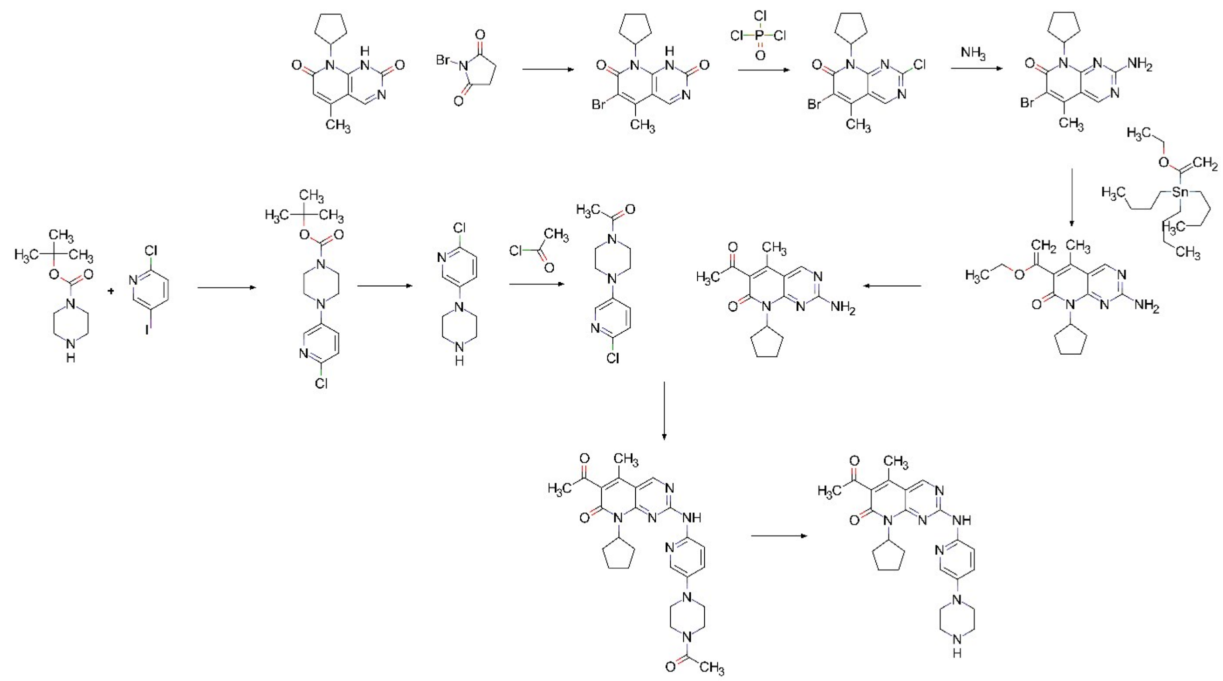

Compound: Tenofovir Alafenamide

Unsolved: 25.33 seconds

Reason: Precursor not in stock

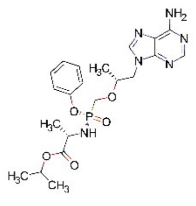

25.33127474784851

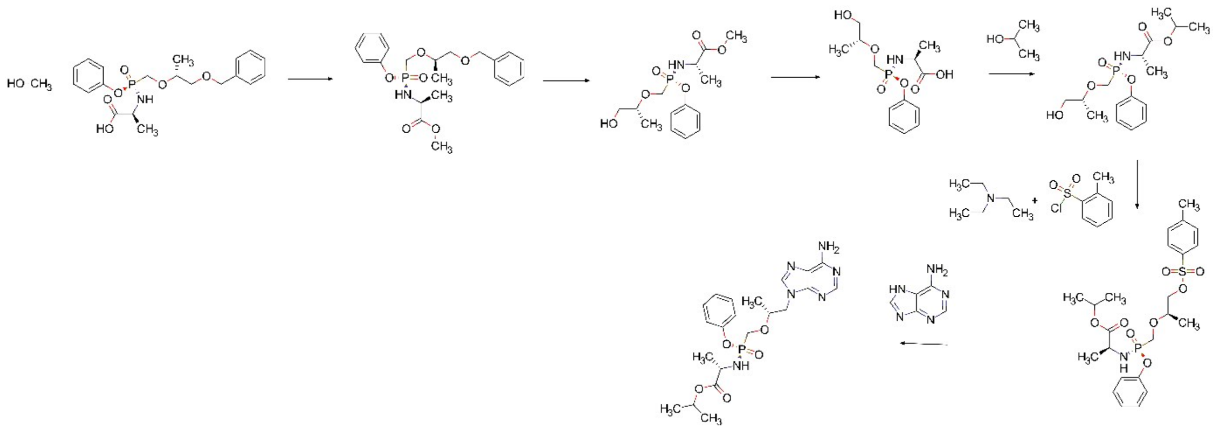

Supplement: Supplementary file 1 [file SC-011-C9SC04944D-s001.pdf]
